# Increased mitochondrial DNA diversity in ancient Columbia River basin Chinook salmon *Oncorhynchus tshawytscha*

**DOI:** 10.1371/journal.pone.0190059

**Published:** 2018-01-10

**Authors:** Bobbi M. Johnson, Brian M. Kemp, Gary H. Thorgaard

**Affiliations:** 1 School of Biological Sciences, Washington State University, Pullman, Washington, United States of America; 2 Department of Anthropology, Washington State University, Pullman, Washington, United States of America; National Cheng Kung University, TAIWAN

## Abstract

The Columbia River and its tributaries provide essential spawning and rearing habitat for many salmonid species, including Chinook salmon (*Oncorhynchus tshawytscha*). Chinook salmon were historically abundant throughout the basin and Native Americans in the region relied heavily on these fish for thousands of years. Following the arrival of Europeans in the 1800s, salmon in the basin experienced broad declines linked to overfishing, water diversion projects, habitat destruction, connectivity reduction, introgression with hatchery-origin fish, and hydropower development. Despite historical abundance, many native salmonids are now at risk of extinction. Research and management related to Chinook salmon is usually explored under what are termed “the four H’s”: habitat, harvest, hatcheries, and hydropower; here we explore a fifth H, *history*. Patterns of prehistoric and contemporary mitochondrial DNA variation from Chinook salmon were analyzed to characterize and compare population genetic diversity prior to recent alterations and, thus, elucidate a deeper history for this species. A total of 346 ancient and 366 contemporary samples were processed during this study. Species was determined for 130 of the ancient samples and control region haplotypes of 84 of these were sequenced. Diversity estimates from these 84 ancient Chinook salmon were compared to 379 contemporary samples. Our analysis provides the first direct measure of reduced genetic diversity for Chinook salmon from the ancient to the contemporary period, as measured both in direct loss of mitochondrial haplotypes and reductions in haplotype and nucleotide diversity. However, these losses do not appear equal across the basin, with higher losses of diversity in the mid-Columbia than in the Snake subbasin. The results are unexpected, as the two groups were predicted to share a common history as parts of the larger Columbia River Basin, and instead indicate that Chinook salmon in these subbasins may have divergent demographic histories.

## Introduction

Chinook salmon are the largest, and perhaps most well-known, of the Pacific salmon (*Oncorhynchus spp*.). Hatched and reared in freshwater, young fish migrate to the ocean to mature and grow for two to six years before returning to their natal location for spawning and subsequent mortality. Chinook salmon exhibit a wide variety of life histories that are intimately linked to environmental variables such as temperature, photoperiod, and stream discharge [1, 2]. Two generalized forms have evolved, often termed races [3]. The *ocean-type* race limits its time in freshwater, migrating to the ocean shortly after hatching and delaying their return until shortly before spawning. The *stream-type* race spends a greater proportion of their lives in freshwater during both the juvenile and adult portions of the life cycle. Chinook salmon also display seasonal variability in migrations, with runs returning from the ocean in all four seasons. The runs are named for the season of freshwater reentry which initiates the spawning migration.

Chinook salmon have a native distribution in the North Pacific Ocean, ranging from northern Japan and north-east Siberia south to California. The Pacific Northwest provides essential habitat in the species range. Native American utilization of Chinook salmon, along with other salmonids, has been documented in the region for over 9000 years [4]. Fishing tended to center around natural barriers, most commonly waterfalls, which concentrated runs as fish attempted to navigate upriver [5]. Prominent Columbia River falls fishing sites such as Celilo Falls and Kettle Falls were so productive that several thousand Native Americans may have been present at one location over the course of a fish run [6]. Moreover, on the sandy shoals of the lower river, large seines were used to capture fish and, in smaller tributaries, weirs were commonly constructed to guide fish into small baskets or bins for collection [7].

European settlers were quick to recognize the economic opportunity of these extensive salmon runs. Commercial exploitation of Chinook salmon in the Columbia River system can be broadly segregated into four major phases: (1) 1866 to 1888: initial development of the fishery, (2) 1889 to 1922: the productive phase, (3) 1923 to 1958: period of notable decline, (4) 1958 to current: maintenance of reduced productivity [8]. During the productive phase as many as 11 million kg of Chinook salmon were harvested annually. The Chinook salmon harvest was reduced to 6.8 million kg during the decline and is now maintained at around 2 million kg [8]. Hatcheries were viewed as a means to mitigate the losses and increase production. By the 1905, 62 million eggs and fry were released by hatcheries in the Pacific Northwest [9]. Hatchery mitigation in the Columbia River intensified in the 1960s and by 1995 as much as 80% of the Columbia River Chinook salmon were hatchery-origin fish [8].

**Fig 1:**
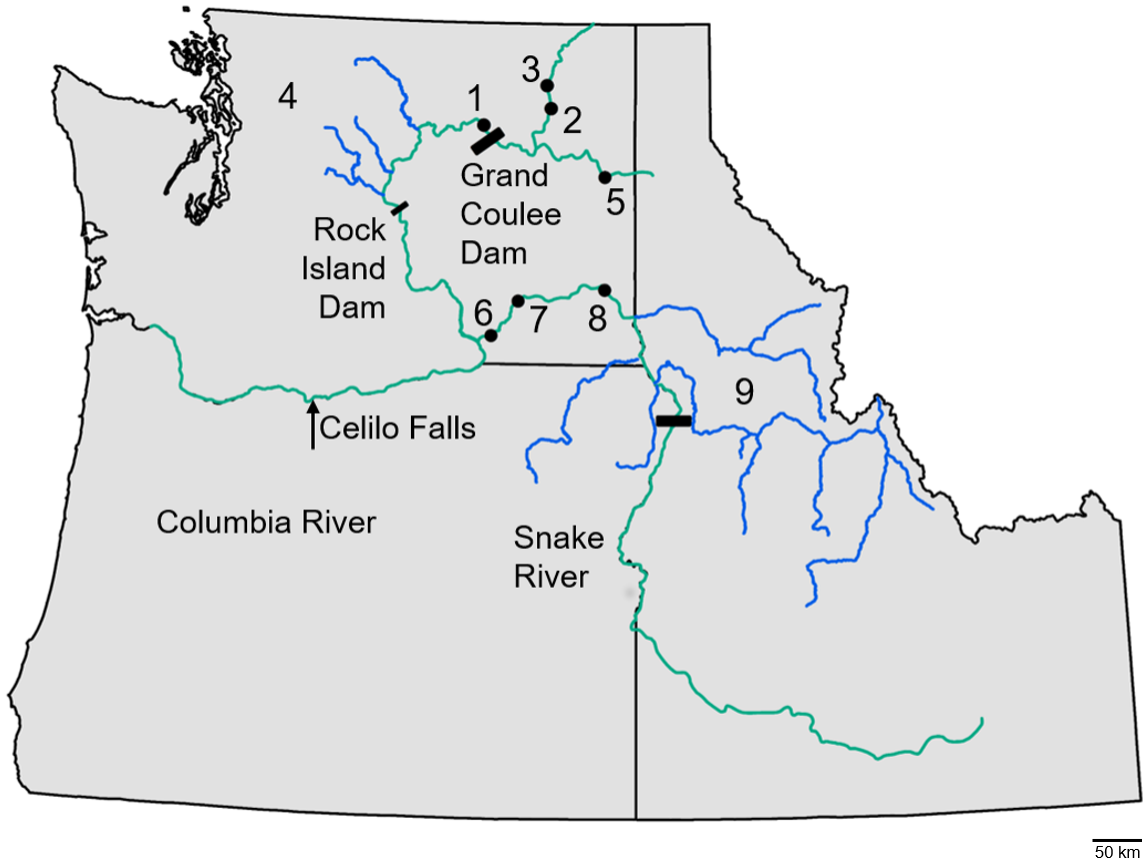
Locations discussed in the study. Ancient sample locations for the Columbia (1–3), Snake (6–8), and Spokane (5) sample groups are indicated with black circles. Contemporary sample locations for the Columbia (near 4) and Snake (near 9) are indicated with blue lines.

Further threats to salmon followed construction of the first mainstem dam on the Columbia River at Rock Island in 1933 (Fig 1). Construction of Bonneville Dam began the same year, with completion in 1938. This was followed by the construction of Grand Coulee Dam in 1941. Grand Coulee Dam had the greatest impact, blocking anadromous salmonids from 1770 river kilometers (rkm) of the upper-Columbia, approximately 40% of their historically available habitat [7, 10]. Hydroelectric development in the Snake River tributary followed a decade later. Dams were constructed on the lower portion of the river (i.e., Ice Harbor, Lower Monumental, Little Goose, and Lower Granite Dams) from 1956 to 1975. These lower river dams included at least some fish passage for Chinook salmon. However, the Hells Canyon Dam, located on the upper Snake and completed in 1967, did not.

The Columbia River Basin river system is now one of the most hydroelectrically-developed in world [11]. Over four hundred dams can be found in the Columbia River Basin, 56 of which were constructed exclusively for hydropower. Today there are nine dams between the furthest inland salmon spawning tributaries in the mid-Columbia and the ocean, and eight dams between the furthest inland salmon spawning Snake River tributaries and the ocean. In total, more than 55% of the historically available spawning habitat in the Columbia River Basin is now blocked by dams [12].

### Study overview and objectives

Until recently, no methods existed to empirically investigate or quantify historical changes in genetic diversity. Instead, genetic histories were extrapolated from data provided by contemporary samples alone. Methodological and technological advances now permit the study of genetic material extracted from ancient specimens, allowing for more direct characterizations of past dynamics [13–16]. The inclusion of genetic information from ancient samples has confirmed large losses in diversity for many species including musk ox (*Ovibos moschatus*) [17], rodents (*Microtus longicaudus* and *Ctenomys sociabilis*)[18, 19], antelope (*Saiga tatarica*) [20] and sea otter (*Enhydra lutris)* [21], among others. However, other investigations have revealed unexpected results. In a study of the genetic history of Scandinavian bears (*Ursus arcto*), Bray et al. [22] found that low genetic diversity in contemporary populations was similar to that in ancient populations and thus, not the result of demographic declines linked to population bottlenecks as previously hypothesized. While most of the current ancient DNA (aDNA) studies have focused on mammals [17, 20, 23–27], the limited number of studies that have incorporated fish species tend to focus on taxonomic identification [28–34]. However, a few select studies have attempted to map demographic changes in fish species as a response to environmental changes [35, 36]. Most of these studies were focused on the deep past, mapping environmental and climate fluctuations in the Pleistocene for brown trout (*Salmo trutta*) [35] and Atlantic salmon (*Salmo salar*) [37] and the upper Paleolithic period for North Iberian salmonids (*Salmo spp*.) [38].

Our study focuses on the more recent Holocene epoch, a period of population decline for many species [39]. Reductions of census size can lead to lower survival, lower reproductive success, and increased inbreeding which erodes genetic diversity [40]. As previously summarized, large-scale declines of Chinook salmon are well-documented following the early 19^th^ century arrival of Europeans in the Pacific Northwest. Although it is often hypothesized that losses in genetic diversity should be coincident with losses in census sizes [10], no direct quantification of diversity has been made of Chinook salmon from the pre-contact era to enable such a comparison. We sought to directly test the hypothesis that genetic diversity was higher in the pre-contact period relative to the post-contact (contemporary) period. To this end, our objectives were to (1) characterize the mtDNA diversity in ancient Chinook salmon, (2) to compare those patterns to analogous ones from contemporary groups, (3) to quantify changes in diversity, if changes occurred at all, and (4) to broadly explore demographic scenarios that may explain temporal and geographic patterns of diversity. Additionally, we use the data generated to test the hypothesis that haplotypes currently found in warmer (southern) and/or cooler (northern) portions of the species range are associated with historical climate periods.

## Methods

### Samples

The research presented here focuses on three complementary sample groups of archeological sites in the Columbia River Basin referred to as the: (1) Columbia River group, (2) Snake River group, and (3) Spokane River group (Fig 1). The three sample groups share a lower river connection to the ocean and would have experienced broadly similar climatic and geological events. Both the ancient Columbia and Snake River samples represent fish caught by Native Americans as the salmon migrated up the mainstem portion of the respective rivers to a number of terminal spawning locations. These groups can be thought of as “mixed stock” sample collections. In contrast, samples in the Spokane group were collected at a terminal fishing location a short distance below an impassable falls rather than a site where multiple local stream populations would be passing [41]. It is likely that the remains found at this site represent fish that were returning to spawn very near the location and the Spokane group can be thought of as a “single stock”.

There are various definitions for subdividing the regions of the Basin; notable are definitions of the mid-Columbia and upper-Columbia subbasins. For the purposes of this study we use *lower* to refer to the area downstream of the Columbia-Snake confluence, *mid* for area from the Snake-Columbia confluence to the Grand Coulee Dam and *upper* for the area above the Grand Coulee Dam (Fig 1). Although the upper-Columbia historically supported many anadromous fish populations, the area we refer to as the mid-Columbia now represents the uppermost spawning habitat for these life histories.

A total of 712 ancient and contemporary samples were processed for this study. Ancient samples consisted of a total of 346 vertebrae (S1 Fig and S2 Table), recovered from middens (i.e., ancient garbage piles), previously classified in their respective collections as “*Oncorhynchus spp*.” or “likely *Oncorhynchus spp*.” based on visual analysis. Contemporary samples consisted of 366 fin clips, stored in ethanol, taken from Chinook salmon (Table 1 and S1 Table). The data obtained from these directly extracted and sequenced samples was supplemented with those of Martin et al. [42] in their survey of Chinook salmon throughout the species range. Samples are summarized by group below and specific contextual descriptions are provided in S1 Text.

**Table 1:** Haplotypes sampled, richness (HR), richness adjusted to smallest sample size of 24 (HRADJ), as well as haplotype (h) and nucleotide (π) diversity. Data indicates contemporary samples have limited genetic diversity relative to ancient counterparts across all metrics for all sample groups.

Group/sub-group (approx. age YBP)	N	Haplotype: TSA___															HR	HR_ADJ_ (SE)	h (var.)	π x100
		1a	1b	4a	10	12	17	18	19	22	23	24	25	26	27	28				
Spokane River Group	26	2	--	--	10	--	9	--	--	--	--	--	3	--	1	1	6	6 (0.5)	0.52 (0.004)	0.186
(2500)	2	1	--	--	1	--	--	--	--	--	--	--	--	--	--	--	2			
(3250)	4	--	--	--	4	--	--	--	--	--	--	--	--	--	--	--	1			
(7200)	11	1	--	--	3	--	6	--	--	--	--	--	--	--	1	--	4		0.55 (0.005)	0.195
(3250 or 7200)	9	--	--	--	2	--	3	--	--	--	--	--	3	--	--	1	4			
Columbia River Group: Ancient	34	3	9	--	6	--	13	--	--	--	2	1	--	--	--	--	6	6 (0.6)	0.73 (0.002)	0.196
Fort Colvile (100)	1	1	--	--	--	--	--	--	--	--	--	--	--	--	--	--	1			
GCDPA Tail Race (3127)	28	2	6	--	6	--	12	--	--	--	1	1	--	--	--	--	6		0.71 (0.002)	0.198
Ksunku: Kettle Falls (1150)	4	--	3	--	--	--	--	--	--	--	1	--	--	--	--	--	2			
Shonitkwu: Kettle Falls (7627)	1	--	--	--	--	--	1	--	--	--	--	--	--	--	--	--	1			
Columbia River Group: Contemporary	240	--	5	--	20	1	208	--	--	6	--	--	--	--	--	--	5	3 (0.8)	0.24 (0.001)	0.080
Carson & Leavenworth Hatch.	55	--	1	--	4	--	50	--	--	--	--	--	--	--	--	--	3	2 (0.6)	0.17 (0.004)	0.055
Entiat	53	--	1	--	10	--	42	--	--	--	--	--	--	--	--	--	3	2 (0.5)	0.34 (0.005)	0.116
Icicle Creek	52	--	1	--	2	--	48	--	--	1	--	--	--	--	--	--	4	3 (0.8)	0.15 (0.004)	0.046
Methow	42	--	--	--	3	--	34	--	--	5	--	--	--	--	--	--	3	3 (0.6)	0.33 (0.007)	0.119
Wenatchee	38	--	2	--	1	1	34	--	--	--	--	--	--	--	--	--	4	3 (0.8)	0.20 (0.007)	0.062
Priest Rapids Hatchery* (non-GCFMP)	22	4	2	--	6	--	9	--	1	--	--	--	--	--	--	--	5		0.75 (0.058)	0.276
Snake River Group: Ancient	24	4	--	--	3	--	12	--	--	--	3	--	--	1	--	--	5	5 (NA)	0.64 (0.005)	0.216
Three Springs Bar (300–3000)	6	--	--	--	1	--	3	--	--	--	1	--	--	--	--	--	3			
Granite Point (1500–2500)	1	--	--	--	1	--	--	--	--	--	--	--	--	--	--	--	1			
Granite Point (2500–5000)	1	1	--	--	--	--	--	--	--	--	--	--	--	--	--	--	1			
Harder site (1450)	3	--	--	--	--	--	1	--	--	--	2	--	--	--	--	--	2			
Hatiuhpuh (500–4000)	2	--	--	--	--	--	1	--	--	--	--	--	--	1	--	--	2			
Wexpusnime (700–1000)	9	2	--	--	1	--	6	--	--	--	--	--	--	--	--	--	3		0.50 (0.016)	0.179
Windust Caves (300–4500)	2	1	--	--	--	--	1	--	--	--	--	--	--	--	--	--	2			
Snake River Group: Contemporary	139	11	12	1	6	--	105	1	1	--	--	--	2	--	--	--	8	4 (0.9)	0.42 (0.003)	0.141
Chamberlain	10	--	--	--	--	--	10	--	--	--	--	--	--	--	--	--	1		0.00 (0.000)	0.000
Grande Ronde	20	3	--	--	--	--	16	--	--	--	--	--	1	--	--	--	3		0.32 (0.012)	0.115
Imnaha River	9	1	1	--	2	--	5	--	--	--	--	--	--	--	--	--	4		0.64 (0.016)	0.189
Lemhi	8	1	--	--	--	--	7	--	--	--	--	--	--	--	--	--	2		0.25 (0.032)	0.089
Lyons Ferry Hatchery**	22	3	11	1	4	--	1	1	1	--	--	--	--	--	--	--	7		0.72 (0.007)	0.300
Middle Fork Salmon R.	17	1	--	--	--	--	16	--	--	--	--	--	--	--	--	--	2		0.12 (0.010)	0.042
South Fork Salmon R.	12	1	--	--	--	--	11	--	--	--	--	--	--	--	--	--	2		0.17 (0.018)	0.059
Tucannon**	21	--	--	--	--	--	21	--	--	--	--	--	--	--	--	--	1		0.00 (0.000)	0.000
Upper Salmon	20	1	--	--	--	--	18	--	--	--	--	--	1	--	--	--	3		0.18 (0.011)	0.065

*Data from Martin et al. [42] not included in contemporary Columbia River summary data.

**Data from Martin et al. [42] not included in contemporary Snake River summary data.

#### Columbia River group

Ancient samples for the Columbia River group are comprised of vertebrae from three locations near or above the current location of the Grand Coulee Dam (locations 1–3 depicted in Fig 1). The ancient complement of the Columbia River Group focuses on spawning aggregates that historically migrated upstream of Grand Coulee Dam. No comparable contemporary samples are available from these upper-Columbia sites due to the construction of Grand Coulee Dam in the 1930s, which abolished salmon passage upstream of its location [10]. However, during construction of the dam, biologists implemented the Grand Coulee Fish Maintenance Project (GCFMP), which attempted to redirect spawning efforts of fish that would naturally pass the dam into downstream tributaries. From 1939 to 1943, fish were captured at Rock Island Dam (see Fig 1) and transported to release points in tributaries (the Wenatchee, Entiat and Methow Rivers) below Grand Coulee or were propagated in hatcheries [43]. The rivers selected for transplant had previously supported large runs of salmonids but had highly depressed populations by the time the transplant operation was initiated [43]. As a result of the effort, subsequent generations of Chinook salmon in the redirection tributaries became a mix of the progeny of the relocated stocks and any fish autochthonous to the tributaries [10]. Contemporary samples in this group are from fish collected from the transplant rivers utilized for the Grand Coulee Fish Maintenance project (location 4 depicted in Fig 1). The contemporary portion of the Columbia River group is comprised of samples collected from the redirection tributaries between 1995 and 2011 (S1 Table). Contextual descriptions are provided in S1 Text.

#### Snake River group

Ancient samples for the Snake River group are comprised of vertebrae from seven excavation locations coinciding with three contemporary dams along the Snake River (locations 6–8 depicted in Fig 1). Sample dating for the Snake River group proved difficult. Many of the collections indicated high levels of disturbance attributed to construction of a nearby railroad line and/or looting of the archaeological sites [44–48]. In these cases, the dates are conservatively provided as a range of possible ages (Table 1 and S2 Table). Contemporary samples in the Snake River group consisted of wild and hatchery origin fish with fall, spring, and summer run timing life histories (location 9 depicted in Fig 1). Locations were targeted to include all stock reporting groups, providing the opportunity to adequately capture the genetic diversity in the Snake subbasin today [49]. Contextual descriptions are provided in S1 Text.

#### Spokane River group

The third sample group originated from a collection of materials excavated near the Spokane River (location 5 depicted in Fig 1). The location was an ancient fishing site used for over 7000 years [50, 51]. Contextual data for the samples is provided in S1 Text. Anadromous fish have been extirpated from the Spokane River since the 1930s, leaving no directly comparable contemporary counterpart for this group. Throughout this study, comparisons are made between the ancient Spokane River group and the contemporary Columbia River subgroups. While not directly connected, the intent is to compare them as single-stock components of Chinook salmon, to ones sampled from proximate geographic locations in the Columbia Basin.

#### Ethics statement

No tissue (contemporary) or archeological (ancient) samples were directly collected for this study. All samples were provided by external agencies and groups (See S1 Table and S1 Text). As such, n*o permits or institutional oversight by the* Institutional Animal Care and Use Committee (IACUC) or equivalent ethics committee(s) were *required for the described study*, *which complied with all relevant regulations*.

### DNA extraction and amplification

The ancient DNA work described here was performed in the dedicated ancient DNA lab at Washington State University. Prior to destructive analysis, sample weight was collected for all vertebrae, including partial samples. DNA extractions were then attempted and the extracts systematically tested for inhibition and amplification following Kemp et al. [52]. Following amplification, species was confirmed using a 148 bp mtDNA sequences from the 12S region of the genome [53].

Haplotypes were based on a 563 bp sequence, corresponding to bp 573–1135 of the complete reference sequence (NCBI accession NC_002980), which included 414 base pairs (bp) of the 3’ end of the ‘control region, the complete phenylalanine tRNA gene, and 81 bp of the 5’ end of the 12S ribosomal RNA gene. The sequenced region was homologous to that previously sequenced from Chinook salmon throughout their range [42]. Processing of contemporary samples consisted of DNA extraction, amplification, and haplotype determination. Detailed methodology for ancient and contemporary samples is described in S2 Text.

For both the 12S and control region results, all new or rare (those observed from less-than three samples) haplotypes, any sequences with multiple peaks, as well as a random sample (31%) of all results were confirmed via repeated PCR amplification and sequencing of the template [54].

### Genetic diversity and differentiation

Haplotype richness (the number of haplotypes sampled) was drawn directly from the data. However, the final number of haplotyped individuals varied between the contemporary and ancient sample components (7:1 for the Columbia River group and 6:1 for the Snake River group). To account for sampling variance, rarefaction curves [55, 56] were generated for the Columbia, Snake, and Spokane samples. Rarefaction curves were generated using Vegan [57] in program R v.3.3.1 [58]. An adjusted value of haplotype richness (HR_ADJ_), based on the smallest sample size, was taken from the rarefaction data. The data was plotted with SigmaPlot v.12.0 (Systat Software, San Jose, CA) to allow graphical comparison of ancient and contemporary data sets.

Haplotype (h) and nucleotide (π) diversity [59] were calculated using DNAsp [60]. Samples were grouped spatially by subbasin (i.e., Columbia subbasin, Snake subbasin) for analysis (Table 1). The single, intermediate Fort Colvile sample was excluded when calculating genetic diversity for pooled ancient and contemporary groupings.

**Fig 2:**
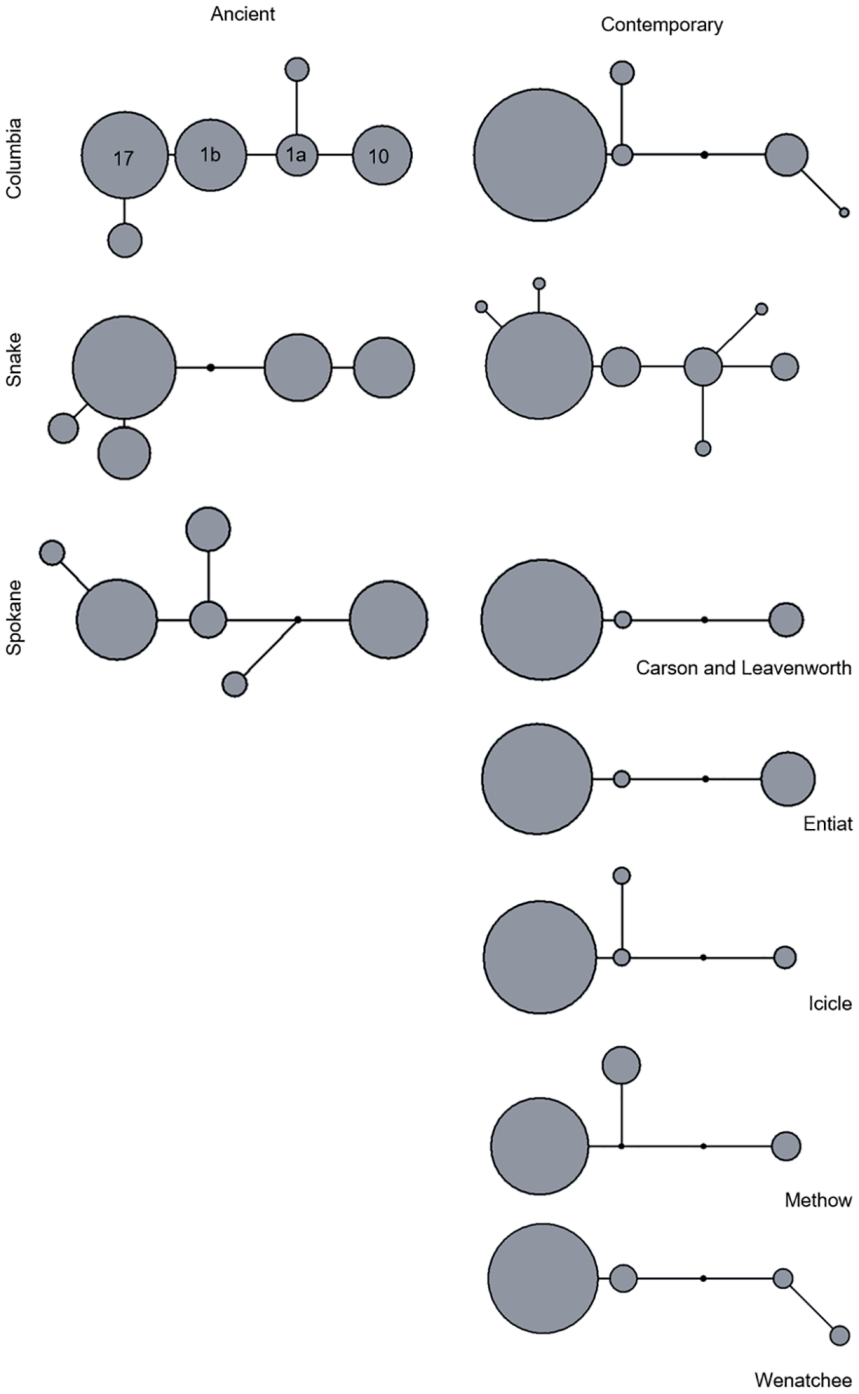
Haplotype networks of ancient and contemporary groups, including Columbia sub-groups. Orientation for haplotypes is constant between networks, circle size is proportional to frequency in the grouping, lines represent mutational connections. Four “evolutionary backbone” types as defined by Martin et al. [42]are labeled for reference in the network.

**Fig 3:**
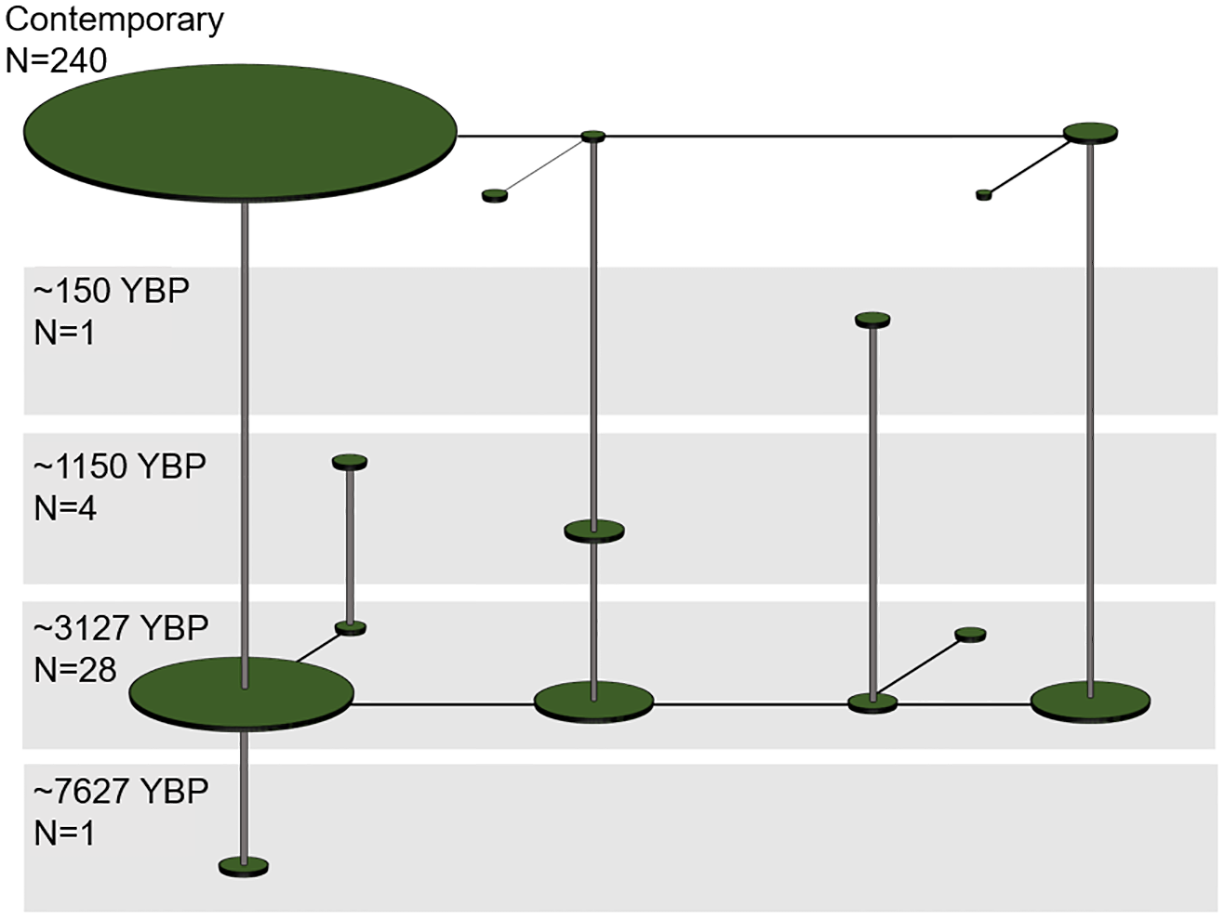
Three-dimensional, temporal haplotype network for all Columbia River samples. Circle sizes are proportional to relative haplotype frequencies, horizontal lines represent single mutation connections between haplotypes.

Haplotype networks were constructed in program Network version 4.613 [61]. Character weights were set to 20 for indels and 30 for transversions to account for the rarity of such events. Transitions and other characters were left at default weight of ten. Three-dimensional haplotype networks were created as described here, and then reproduced by hand using graphical software. The single Fort Colvile sample was excluded from the ancient and contemporary networks (Fig 2), but was included in the three-dimensional network (Fig 3).

The extent of genetic differentiation between ancient and contemporary subgroups was investigated with pairwise comparisons (φ_ST_) [62, 63] and modified exact tests of population differentiation [64]. The intermediate Fort Colvile sample was excluded from both analyses. Values of pairwise φ_ST_ were calculated in Arlequin [65]. Five-thousand permutations of haplotypes between populations was used to test the null hypothesis of no difference between the defined populations [62]. Quartiles of low, moderate, high, and very high differentiation were defined directly from the data. Exact tests were implemented in Arlequin [65] using 10,000 Markov chain steps and 1,000 dememorization steps. The null hypothesis of panmixia based on expected vs. observed haplotype frequencies [64, 66] was evaluated using significance at the 0.05 level of probability.

### Phylogenetic analysis

The survey of Chinook salmon mtDNA haplotypes provided by Martin et al. [42] indicated that distinct haplotypes are present in the northern (Alaska and Kamchatka, Russia) and southern (California) portions of their range. To explore if any of the novel types sampled here fit with the northern or southern clades, we generated a Bayesian cladogram comparing them to the haplotypes sampled by Martin et al. [42]. The phylogeny was generated using MrBayes 3.2 [67]. By default, this program ignores positions that include gaps (indels) so these were coded as binary characters and included in the analysis. We used the General Time Reversible (GTR) model with a proportion of invariable sites (I), and gamma-shaped (Γ) distribution of rates across sites, as determined as the best fitting model using jModelTest [68]. The program was run for 1,000,000 generations and sampled every 100^th^ generation. To ensure that the sampling was taken from a stationary posterior distribution, two independent, simultaneous runs were completed and the standard deviation of the runs compared. The first 25% of runs were discarded as burn-in, with sampling taken from the remaining runs.

### Demographic history

Demographic history was explored via a coalescent model, implemented in BEAST v. 1.8.3 [69]. Two demographic models (1) constant population size [70] and (2) extended Bayesian skyline plot (EBSP) [71] were applied to the Columbia River Group the Snake River group. Final analyses were performed with the GTR + I + Γ model of sequence evolution under a strict molecular clock with a mutation rate of 7.5E-9 to 1.0E-8. This rate was based on that proposed for the mitochondrial control region of salmonids by Shedlock et al. [72] and Thomas et al. [73]. Population size was modeled with a lognormal distribution with initial value of 10,000, mean 5.0, and standard deviation 2.0. Ages for ancient samples were entered as the radiocarbon date and its associated error. In the case of the Snake River samples, where only a range of dates are available, the full range was included in the model. Markov chains were run for 5 X 10^8^ generations (10% discarded as burn-in), sampled every 5 X 10^4^ generations which resulted in a final posterior of 10,000 sampled genealogies. Quality of the posterior distribution was evaluated using the effective sample size (ESS) [74] following Kuhner [75]. Comparisons of models utilized Bayes factor (BF) as determined by differences in marginal likelihood estimates (MLE) [76, 77] described qualitatively using the framework provided in Kass and Raftery [78]. ESS and MLE were calculated in Tracer v.1.6 [69]. Final model parameters were based on comparison of independent exploratory trials. For the final models, two independent runs of each were performed and posterior estimates taken from the combined Markov chains. EBSPs were plotted to ~10,000 YBP using SigmaPlot v.12.0 (Systat Software, San Jose, CA).

## Results

A total of 346 ancient and 366 contemporary samples were processed during this study. Species determination was possible for 130 ancient samples with the 12S marker and sequencing of the control region haplotypes for 84 of these 130. In the Spokane and Columbia River groups, all samples successfully sequenced for the 12S region (species identification) were confirmed to be Chinook salmon. In the ancient Snake River samples, 24 of the 50 samples successfully sequenced for the 12S region were confirmed as Chinook salmon. The remaining 26 included two coho salmon (*Oncorhynchus kisutch)*, sixteen suckers (*Catostomus spp*.), and eight pikeminnows (*Ptychocheilus spp*.) (S2 Table). A total of fifteen unique control region haplotypes were sampled in the study (Table 1). All haplotypes were separated by five or fewer mutations (mean = 2.8) (Fig 2). Seven previously unidentified types were sampled, one exclusive to contemporary samples, five exclusive to ancient samples, and one sampled in both ancient and contemporary samples. New types were designated according to the established nomenclature for Chinook salmon [42, 79], TSA22 –TSA28. Sequences are available from the NCBI database with accession numbers KY973380—KY973386. Haplotypes were determined for 336 of the contemporary samples (no 12S identification was necessary as species was determined antemortem).

### Genetic diversity and differentiation

Comparisons of haplotypes indicate a reduction in number and shift towards fixation by a single haplotype from the ancient to the modern samples in the Columbia River group (Fig 2). In contrast, the Snake group had more haplotypes present in the contemporary group than in the ancient. However, after rarefaction adjustment to the smallest sample size (N = 24) the ancient groups had the highest richness values in all groups: 6 (Spokane and Columbia), and 5 (Snake, unadjusted) (Table 1 and S2 Fig). All estimates of haplotype and nucleotide diversity are given in Table 1. In the Columbia River group, greater diversity is indicated for the ancient samples (h = 0.73 and π = 0.196) than for the contemporary samples (h = 0.24 and π = 0.080) as well as for all five subgroups (ranges of h = 0.17 to 0.34 and π = 0.055 to 0.119). In the Snake River group, the diversity in ancient samples (h = 0.64 and π = 0.216) is greater than the contemporary (h = 0.42 and π = 0.141) as well as seven of the nine contemporary subgroups. The Spokane group, representing a single stock of Chinook salmon, had ancient diversity of h = 0.52 and π = 0.186 which is greater than that present in any of the contemporary Columbia subgroups.

The Spokane samples are not significantly differentiated from the ancient Columbia samples, but are *very highly* differentiated from the contemporary Columbia samples (Table 2). The Spokane and Snake were *moderately* differentiated (ancient) and *highly* differentiated (contemporary). The ancient and contemporary Columbia samples were *moderately* differentiated. The ancient Snake River samples were also similar to their contemporary counterpart, with non-significant and *low* differentiation (φ_ST_ = 0.04). Using modified exact tests, the Spokane River sample group was not significantly different from the ancient Columbia samples but was significantly different from the ancient Snake and both the contemporary sample sets (Table 3 and S3 Table). In both the Columbia River and Snake River group, haplotype frequencies for the ancient and contemporary samples were significantly different.

**Table 2:** Pairwise φST for ancient and contemporary sample groupings. Raw φST values are listed above and color-coded by quartile below, underlined values indicate significance (α ≤ 0.05). Non-significant values colored as values of 0 (no differentiation).

	ASP	Columbia									Snake												
		ANC	CON	CPR	CLH	ENT	ICI	MET	WEN	PRH	ANC	CON	CHA	GRO	IMN	LEM	LFH	MFS	SFS	TUC	UPS		
Ancient Spokane (ASP)	xx	0.09	0.48	0.42	0.46	0.26	0.50	0.35	0.43	0.01	0.15	0.37	0.41	0.27	0.03	0.28	0.08	0.40	0.35	0.49	0.36		low
Columbia River group																							moderate
Ancient (ANC)		xx	0.24	0.18	0.25	0.09	0.29	0.15	0.23	0.00	0.02	0.13	0.25	0.10	0.00	0.11	0.04	0.22	0.18	0.31	0.19		high
Contemporary (CON)			xx	0.00	0.00	0.03	0.00	0.01	0.00	0.32	0.11	0.02	0.00	0.02	0.18	0.00	0.48	0.00	0.00	0.03	0.00		very high
Contemporary & Priest Rapids H.* (CPR)				xx	0.00	0.01	0.02	0.00	0.01	0.25	0.06	0.01	0.02	0.00	0.11	0.00	0.41	0.01	0.00	0.05	0.00		
Carson and Leavenworth H. (CLH)					xx	0.04	0.00	0.02	0.00	0.33	0.12	0.02	0.00	0.03	0.24	0.00	0.50	0.00	0.00	0.02	0.00		
Entiat (ENT)						xx	0.07	0.03	0.05	0.12	0.01	0.02	0.07	0.01	0.01	0.00	0.29	0.05	0.02	0.12	0.03		
Icicle (ICI)							xx	0.02	0.00	0.38	0.16	0.04	0.00	0.06	0.32	0.00	0.54	0.00	0.00	0.01	0.00		
Methow (MET)								xx	0.01	0.22	0.06	0.01	0.03	0.01	0.11	0.00	0.35	0.02	0.00	0.07	0.01		
Wenatchee (WEN)									xx	0.31	0.11	0.02	0.00	0.02	0.23	0.00	0.47	0.00	0.00	0.02	0.00		
Priest Rapids H.* [non-GCFMP] (PRH)										xx	0.04	0.21	0.32	0.15	0.00	0.16	0.04	0.30	0.24	0.41	0.25		
Snake River group																							
Ancient (ANC)											xx	0.04	0.13	0.01	0.00	0.00	0.17	0.10	0.06	0.20	0.07		
Contemporary (CON)												xx	0.05	0.00	0.08	0.00	0.35	0.02	0.00	0.08	0.00		
Chamberlain (CHA)													xx	0.08	0.30	0.03	0.48	0.00	0.00	0.00	0.00		
Grande Ronde (GRO)														xx	0.05	0.00	0.30	0.03	0.00	0.15	0.00		
Imnaha River (IMN)															xx	0.07	0.06	0.26	0.17	0.44	0.18		
Lemhi (LEM)																xx	0.33	0.00	0.00	0.13	0.00		
Lyons Ferry H.* (LFH)																	xx	0.46	0.40	0.57	0.41		
MF Salmon R. (MFS)																		xx	0.00	0.01	0.00		
SF Salmon R. (SFS)																			xx	0.05	0.00		
Tucannon (TUC)																				xx	0.05		
Upper Salmon (UPS)																					xx		

*Data from Martin et al. [42].

**Table 3:** Exact tests of population differentiation. Comparisons that differ significantly from that expected under panmixia are indicated with "+", those that cannot be distinguished from a panmictic population with "-".

	ASP	Columbia									Snake										
		ANC	CON	CPR	CLH	ENT	ICI	MET	WEN	PRH	ANC	CON	CHA	GRO	IMN	LEM	LFH	MFS	SFS	TUC	UPS
Ancient Spokane (ASP)		-	+	+	+	+	+	+	+	-	+	+	+	+	-	-	+	+	+	+	+
Columbia River group																					
Ancient (ANC)	-		+	+	+	+	+	+	+	-	+	+	+	+	-	-	+	+	+	+	+
Contemporary (CON)	+	+		-	-	-	-	-	-	+	+	+	-	+	+	-	+	-	-	-	+
Contemporary & Priest Rapids H.* (CPR)	+	+	-		-	-	-	-	-	+	+	+	-	+	-	-	+	-	-	-	-
Carson and Leavenworth H. (CLH)	+	+	-	-		-	-	+	-	+	+	-	-	+	+	-	+	-	-	-	-
Entiat (ENT)	+	+	-	-	-		+	+	+	+	+	+	-	+	-	-	+	-	-	-	+
Icicle (ICI)	+	+	-	-	-	+		-	-	+	+	-	-	+	+	-	+	-	-	-	-
Methow (MET)	+	+	-	-	+	+	-		+	+	+	+	-	+	+	-	+	-	-	-	-
Wenatchee (WEN)	+	+	-	-	-	+	-	+		+	+	-	-	+	+	-	+	-	-	-	-
Priest Rapids H.* [non-GCFMP] (PRH)	-	-	+	+	+	+	+	+	+		-	+	+	+	-	-	+	+	+	+	+
Snake River group																					
Ancient (ANC)	+	+	+	+	+	+	+	+	+	-		+	-	-	-	-	+	-	-	+	+
Contemporary (CON)	+	+	+	+	-	+	-	+	-	+	+		-	-	-	-	+	-	-	-	-
Chamberlain (CHA)	+	+	-	-	-	-	-	-	-	+	-	-		-	+	-	+	-	-	-	-
Grande Ronde (GRO)	+	+	+	+	+	+	+	+	+	+	-	-	-		-	-	+	-	-	+	-
Imnaha River (IMN)	-	-	+	-	+	-	+	+	+	-	-	-	+	-		-	+	+	-	+	+
Lemhi (LEM)	-	-	-	-	-	-	-	-	-	-	-	-	-	-	-		+	-	-	-	-
Lyons Ferry H.* (LFH)	+	+	+	+	+	+	+	+	+	+	+	+	+	+	+	+		+	+	+	+
MF Salmon R. (MFS)	+	+	-	-	-	-	-	-	-	+	-	-	-	-	+	-	+		-	-	-
SF Salmon R. (SFS)	+	+	-	-	-	-	-	-	-	+	-	-	-	-	-	-	+	-		-	-
Tucannon (TUC)	+	+	-	-	-	-	-	-	-	+	+	-	-	+	+	-	+	-	-		-
Upper Salmon (UPS)	+	+	+	-	-	+	-	-	-	+	+	-	-	-	+	-	+	-	-	-	

*Data from Martin et al. [42].

### Phylogenetic analysis of haplotypes

**Fig 4:**
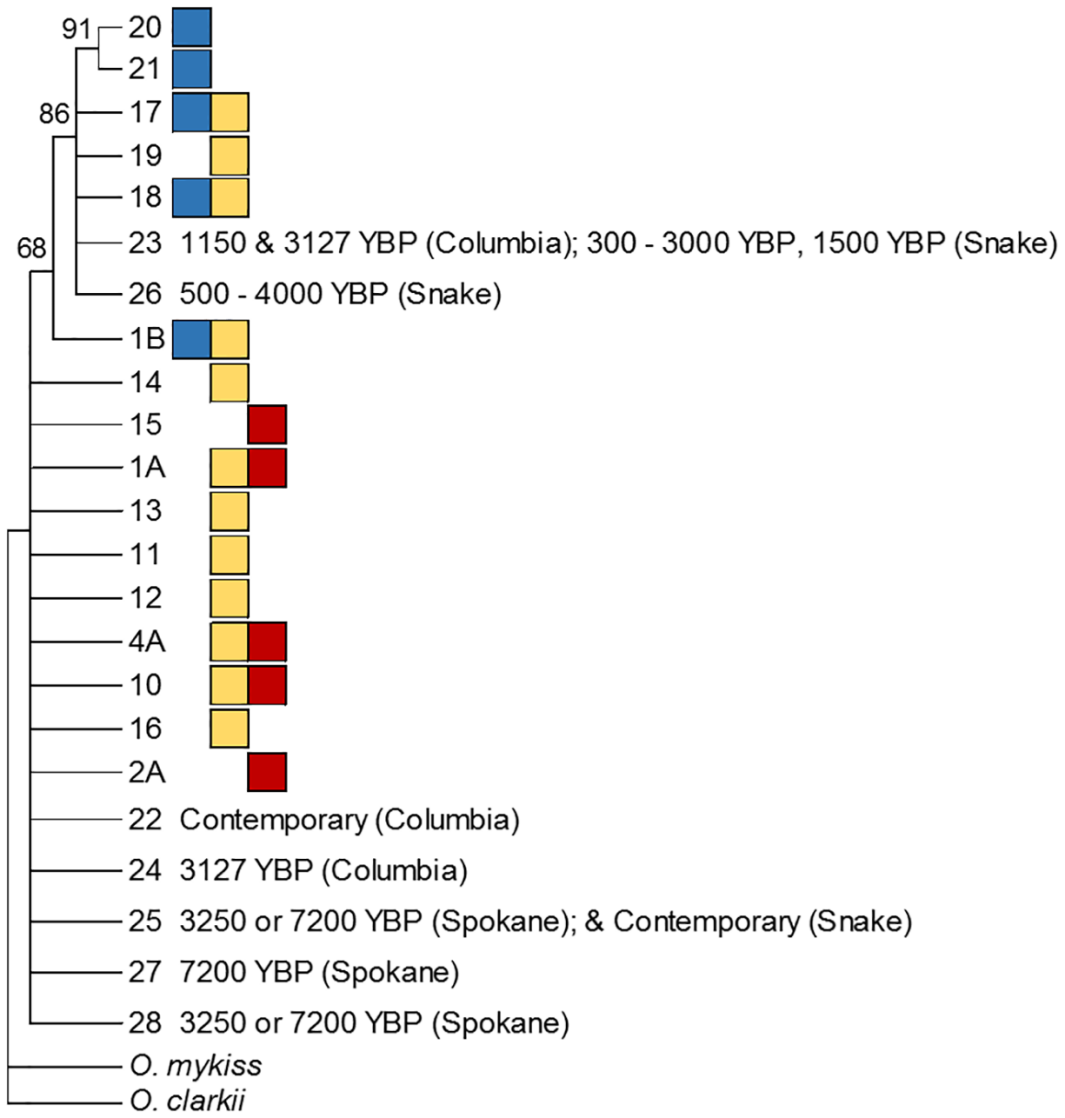
Bayesian cladogram for Chinook haplotypes from full species range, as well as novel haplotypes detected in this study. Previously identified types are coded according to geographic region north: blue, central: yellow, southern: red per designations in Martin et al. [42]. All novel types fell into shared geographic clades (Northern/Central or Central/Southern).

Seven novel haplotypes were identified in this study. When these were combined with published haplotypes for Chinook salmon [42], two fell into the clade of shared northern and centrally sampled haplotypes and the remaining five into the clade of shared central and southerly sampled haplotypes (Fig 4).

### Demographic history

**Fig 5:**
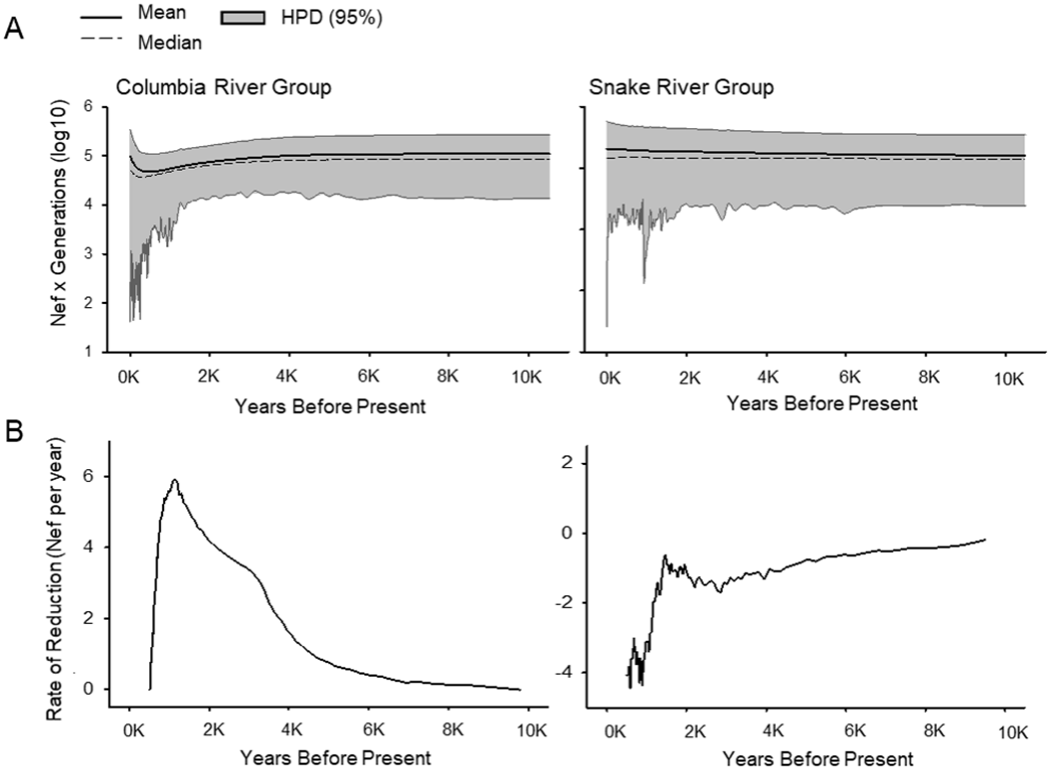
Extended Bayesian skyline plots (EBSPs) for Columbia and Snake River groups (A) and rate of reduction in Nef indicated in the associated EBSP (B). Mean effective population size is indicated with solid black line, median with dashed line, and 95% highest posterior density (HPD) in shaded grey. Contrasting patterns are present for the two groups; the Columbia River groups visually indicates a reduction in mean effective population size while no such reduction is present for Snake River samples.

Four total models were implemented in BEAST (Table 4) which compared a model of constant population size to a demographic EBSP model for Columbia and Snake River groups. Note, here *population* refers to the temporal subgroups ancient Columbia, contemporary Columbia, ancient Snake, and contemporary Snake. When applied to the Columbia River sample data, the demographic (EBSP) model supported over that of constant population size and the support level qualified as “strong” (9.38) [74, 75]. For the Snake River samples, the constant population size and EBSP models indicated relatively equal support (“not worth more than a bare mention” BF 0.65) [75]. When plotted, the EBSP for the Columbia River group shows some variation in effective population size overtime, such a pattern is not present in the Snake River (Fig 5).

**Table 4:** Summary of demographic models implemented in BEAST including effective sample sizes (ESS). Comparisons are given as the difference in likelihood, positive values indicate a better fit of the demographic model (B) compared to the model of constant population size model (A).

Description			Model	ESS	Bayes factor model comparison
1	A	Columbia: Ancient and contemporary	Constant	391	--
	B	Columbia: Ancient and contemporary	EBSP	207	9.38
2	A	Snake: Ancient and contemporary	Constant	3392	--
	B	Snake: Ancient and contemporary	EBSP	3665	0.65

## Discussion

Our results reveal contemporary Chinook salmon in parts of the Columbia River basin are genetically depauperate relative to their ancient counterparts. However, the comparisons for genetic diversity were not uniformly distributed through the basin and distinct patterns were present for the groups examined here. Based on our data, Chinook populations in the upper-Columbia may have experienced larger losses in genetic diversity than those in the Snake River. The index of φ_ST_ can be connected to migration, specifically that migration erodes φ_ST_ [80]. In comparisons of the ancient and contemporary subgroups, *migrants* are actually *temporal migrants*, genetic variants persisting through time, instead of traveling through space. Few such *temporal* migrants appear to be present between the ancient and contemporary components, as indicated by high levels of differentiation (Tables 2 and 3).

The Snake River sample group also indicates a reduction in genetic diversity from the ancient to the contemporary period. However, the losses in genetic diversity were of less magnitude than that observed in the Columbia group. In contrast to the Columbia River group, more haplotypes were observed in the contemporary samples than in the ancient and the rarified comparisons are very similar (Table 1). Diversity comparisons indicate approximately one-third less diversity (for both h and π) in the contemporary samples than in the ancient. The ancient and contemporary Snake samples show only low, non-significant differentiation (Table 2), indicative of temporal continuity in this group.

The Columbia and Snake River groups also differed in the demographic analysis. No evidence for a population decline was identified for the Snake River samples and a model of constant population size was equally supported when compared to the demographic model. In contrast, the analysis of the Columbia River samples lends support for the demographic model with a reduction in effective population size.

Coalescent analyses are powerful tools to elucidate historical demography but are not without limits. As with conventional population statistics, undefined population structure in coalescent analysis can lead to erroneous estimates of demographic history [81–84]. The extent and potential impact of population structure on our dataset is difficult to infer. Salmon runs in the Pacific Northwest were established within the past 10,000 years [4] and any population structure would have been subsequently developed. All recolonization was accomplished via straying [85, 86], which may have limited population structure. Further, during this period the region was subject to climatic and geological disturbance [87–90] which may have eroded existing structure, if present at all. The ancient Spokane samples are a single-stock component of the mixed-stock Columbia River group, providing some data from which to test the hypothesis of panmixia. Differentiation between the Spokane and the larger Columbia group is low (0.09) (Table 2) and haplotype frequencies did not differ significantly from that expected if the groups were panmictic (Table 3). Differentiation analysis of contemporary subgroups in our study indicates that contemporary subpopulations are not highly differentiated from each other either (Tables 2 and 3). However, contemporary data has likely been influenced to some degree by management practices and the cumulative impact of 200 years of such practices is not entirely predictable.

Skyline methods are also often limited in their ability to detect recent events. Catastrophic census declines for Columbia basin Chinook salmon were documented during the mid-19^th^ century. It is difficult to imagine that these declines were without genetic consequence. Yet, no evidence for a coincident decline in genetic diversity is indicated in the EBSP for either the Columbia or the Snake data. Even with heterochronous data, recent events are not always detectable, especially when genetic losses are brief, extreme, or occur very near the sampling events [91, 92]. Further, our analysis is based on a uniparentally inherited marker (mtDNA), which can only provide a limited picture of evolution. Future investigations utilizing additional markers may contribute to increased resolution. However, we cannot exclude the possibility that the demographic patterns indicated are true representations of recent history. Examples of sustained diversity, despite periods of largescale population declines, have been demonstrated. For example, Hawaiian petrel (*Pterodroma sandwichensis*) populations were so reduced in numbers during the 1900s that many believed the species to be extinct [93, 94]. However, comparisons of ancient and contemporary DNA revealed limited losses in genetic diversity and maintenance of effective population size through the period of population decline and recovery [92]. Despite the potential limitations, the analysis of the EBSP as a qualitative heuristic for model rejection fits with the larger patterns for the Columbia and Snake River sample groups and provides evidence of contrasting patterns.

The contrasting patterns displayed for the Columbia and Snake River samples are unexpected. The two groups are parts of the larger Columbia River Basin and were predicted to largely share a common history. However, our results indicate that distinct demographic differences for the Columbia and Snake River samples are present. We hypothesize that these differences may be the result of fine-scale differences that extend into the pre-contact era. Here we summarize some potential historical differences for the two groups which support the hypothesis that both pre- and post-contact anthropological impacts may have resulted in the genetic patterns observed.

Prior to contact, Native Americans captured Chinook salmon from both the Columbia and Snake rivers. However, the cumulative intensity of fishing may have been higher for mid-Columbia runs than for Snake River runs. Both the Snake and upper-Columbia River spawning aggregates investigated in this study would have been exploited at Celilo Falls (Fig 1) as well as any additional fishing locations between the ocean and their spawning grounds. However, the upper-Columbia aggregates experienced the additional, formidable pressure of the Kettle Falls fishery and potentially the Spokane Falls fishery. It is estimated that the Colville Tribe alone took as much as 270,000 kg of salmon from Kettle Falls in a single year [95–97]. The Snake had many quality fishing sites but lacked mainstem falls capture locations matching the scale of Kettle or Spokane Falls [97, 98]. Although salmon constituted an important food source, for some Snake River tribes this catch had to be substantially supplemented with other hunting and gathering activities [99]. Non-salmonid fish alternatives may have also been more readily available in the Snake River. White sturgeon (Acipenser transmontanus), a species commonly reaching 20–295 kg likely formed a substantial component of the diet for Snake River tribes as documented for the Nez Perce [97]. Other smaller fish species were also heavily exploited [97] which was consistent with the identification of suckers (Catostomus spp.) and pikeminnows (Ptychocheilus spp.) among the Snake River vertebrae in this study.

Differences in the exploitation realized by upper-Columbia and Snake River stocks continued beyond the prehistoric fishing period. The commercial fishery that developed shortly post-contact was concentrated on the lower river, impacting both upper-Columbia and Snake River stocks. Though commercial fishery operations were primarily conducted in the shared lower river, their impact on the upper subbasins was likely unequal, with increased pressure on mid and upper-Columbia River aggregates relative to those in the Snake River. Stocks containing large Chinook salmon were reduced faster than those with smaller counterparts [100, 101]. Fish migrating to the upper-Columbia were commonly 14 to 36 kg, whereas lower river stocks may have been considerably smaller [41]. The targeting of larger fish depleted upper-Columbia runs faster than lower regions of the Columbia River Basin.

Run timing may have also been a factor for differences in post-contact exploitation. Fall-run Chinook salmon arrive in freshwater in a more advanced spawning condition, making spring and summer-run fish more commercially desirable. Accordingly, the early commercial Chinook salmon fishery focused on spring and summer runs. In the 1880s canneries closed in late July, effectively forgoing any opportunity to capture fall-run Chinook salmon [7]. As salmon populations declined, commercial exploitation shifted to include fall runs by the early 1920s [7, 102]. Fall runs were prevalent in the Snake River and lower reaches of the Columbia River. However, longer migrations and differences in mainstem river characteristics likely limited historic upper-Columbia fall run sizes [41].

Although fall-run Chinook salmon represented a smaller portion of total stocks in the Columbia River, these fish may represent an important component of diversity within the Basin. In the interior Columbia basin, all fall-run and all spring-run Chinook salmon populations fall into two distinct genetic lineages [87]. The divergence of these lineages is believed to have occurred during the Pleistocene [103, 104]. Distinct spawning times likely act to maintain reproductive isolation between the groups, allowing for the accumulation of genetic differences via drift. Evidence for historical inclusion of fall-run Chinook salmon in native fisheries is strong [105]. Fall run fish are likely to be represented in the ancient Columbia River sample group. Site 45DO189, from which 28 samples were studied, was occupied during the fall and potentially winter by Native Americans [106]. Fall Chinook salmon spawn from September to December and are likely to be present in the sample, based on the occupation time. In contrast, no fall-run fish are included in the contemporary Columbia samples. Fall Chinook salmon confine their spawning almost exclusively to mainstem rivers or large tributaries [107] and the GCFMP redirection tributaries were not sufficiently large as to meet the spawning requirements of fall life histories. As a result, contemporary runs of fall Chinook salmon in the Columbia River are almost exclusively confined to a 90 rkm stretch of lower river known as Hanford Reach.

Notably, the ancient Columbia River samples display a number of similarities to contemporary fall-run Chinook salmon from both the Snake River (Lyons Ferry Hatchery) and lower Columbia River (Priest Rapids Hatchery) [42]. Comparisons of haplotype composition, overall genetic diversity, and differentiation (based on both exact tests and φ_ST_) indicate more similarity to the sample of contemporary fall Chinook salmon than to those in the contemporary tributaries used for redirection, particularly to the Chinook salmon from Priest Rapids Hatchery. Genetic components have been indicated for functional life history differences, including run timing in Chinook salmon [108–111]. However, to our knowledge, no functional associations have been made for control region haplotypes, or for mtDNA in general, in Chinook salmon. Previous mtDNA surveys have also indicated quantitative differences in haplotype frequencies between spring/summer and fall run Chinook in the Columbia River [2, 112–114] but a lack of haplotype fixation for either group [114]. It is likely that some haplotypes are more or less common in fall or spring runs due to drift or possibly, yet undetected selection as has been indicated for protein coding segments of the mtDNA genome in salmonids [115]. Summer-run fish were included in the contemporary Columbia samples (N = 19). All but one of the samples from this summer-run subset were monomorphic for haplotype TSA17 and the resulting haplotype diversity was 0.11. In contrast, the fall-run subset of Snake River samples (N = 24) contained seven haplotypes, with a haplotype diversity of 0.72. Based on our data, there is no evidence for summer runs in the upper-Columbia as stores of historic genetic diversity. However, a broader sampling of summer-run fish from the mid-Columbia would be useful to further test this hypothesis.

### Tests for correlation of haplotypes with climatic periods

Climate and stream conditions in the Columbia River Basin have fluctuated dynamically over several millennia and these conditions have been characterized for the past 11,500 years. Published data confirm three macroclimates that overlap our samples: drought conditions with maximum summer warmth (prior to 8000 YBP), transitioning toward moist and cool (8000–4000 YBP), and cool temperatures while slightly drier with late period warming (4000 YBP to contemporary) [88, 89, 116]. Two specific variables impacting salmon life history (water temperature and stream discharge/flow) have also been described. Prior to 5500 YBP flows were ~30% lower than current and freshet ended in June, while from 2300–4500 YBP flows were ~30% higher with freshet ending in August [88].

**Fig 6:**
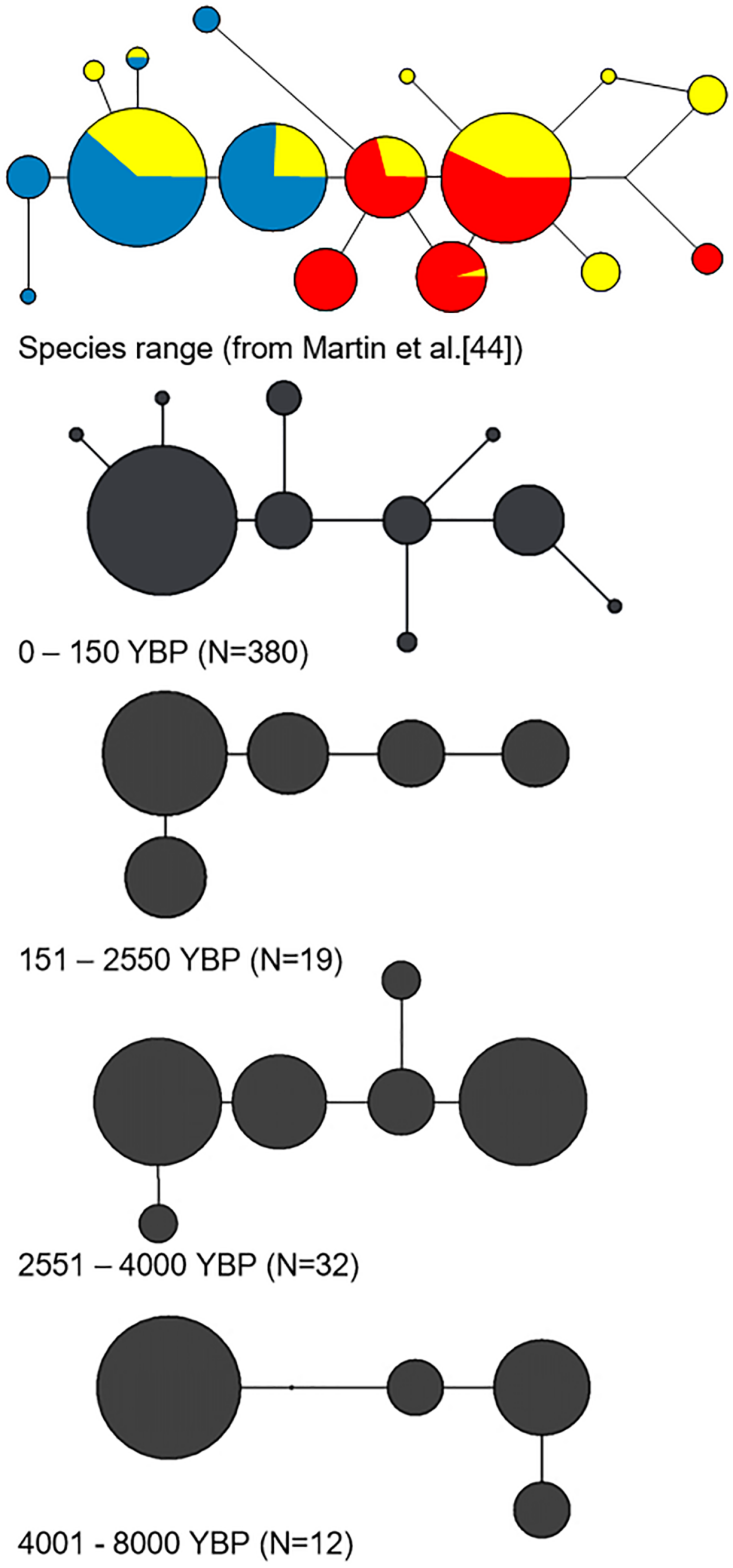
Comparison of spatial (top) and temporal (lower four) haplotype sampling. Orientation for haplotypes is constant between networks, circle size is proportional to frequency in the grouping, lines represent mutational connections. Spatial network (top) is color coded for: southern-red, central-yellow, and northern-blue portions of the species range. No association between haplotypes and climate periods is noted.

Contemporary data on Chinook salmon indicates that of the seventeen published haplotypes, two are found only in northern populations, seven in central populations, and two in only southern populations [42]. Three other haplotypes are shared between northern and central populations and another three between southern and central populations (Fig 6). This phylogeographic distribution is likely a reflection of post-glacial colonization and subsequent genetic drift. However, it is possible that the distribution also reflects selection on functional regions of mitochondrial DNA; evidence for such a phenomenon has recently been described for Pacific salmon [115].

We compared haplotype and phylogeographic characterizations with the available climate data to test the hypothesis that haplotypes currently associated with southern (warmer) regions of the species range coincide with warmer climate periods, and those that associate with northern (cooler) regions with cooler periods. Such a distribution might be anticipated if (1) the mitochondrial haplotypes are correlated with cold or warm-adapted gene complexes in Chinook salmon, (2) the haplotypes served as indicators of southern or northern-adapted nuclear gene complexes, or (3) climate-mediated range shifts occurred in the past. Such range shifts have been demonstrated for many species including birds [117], freshwater fish [118], insects, [119], rodents [120] and plants [121]. The phylogeny of all haplotypes indicated that the haplotypes novel to this study correlated with the central based types (Fig 4) and shared types were present in all temporal climate groupings (Fig 6). We also did not identify any of the distinct northern or southern types in the ancient samples. Our data does not support an association or transition of Chinook salmon haplotypes during varying climate periods and instead, indicates that the distribution of mtDNA lineages has been stable over long periods of time. It is important to note the phylogeny was not well resolved and with only weak support for the clades discussed. Better resolution may be obtained through the inclusion of additional genetic markers.

### Notes on reintroduction

An inter-agency investigation into the feasibility of reintroducing anadromous salmon above the Chief Joseph and Grand Coulee Dams is currently being pursued [122–125]. One element of this evaluation is the identification of candidate fish stocks for use in reintroduction. We caution against extrapolating the data presented here to inferences on successful spawning and recruitment. The comparisons in this study focus on genetic similarity between ancient samples and contemporary groups. However, genetic *similarity* does not necessarily translate to genetic *suitability*. A large body of evidence exists for local adaptation in salmonids [126, 127]. Goals related strictly to levels of genetic diversity ignore the relationship between genes and the environment demonstrated for salmonids.

### Summary

Our results reveal contemporary Chinook salmon in parts of the Columbia River basin are genetically depauperate relative to their ancient counterparts. However, the differences are not uniformly distributed through the basin and distinct patterns are present for the groups examined here. Based on our data, Chinook populations in the upper-Columbia may have experienced larger losses in genetic diversity than those in the Snake River. The contrasting patterns displayed for the Columbia and Snake River samples are unexpected as the two groups were predicted to share a common history as parts of the larger Columbia River Basin. However, each of the groups contains distinct populations of Chinook salmon which may have divergent demographic histories. We hypothesize that these differences may be the result of cumulative effects of pre- and post-contact exploitation along with direct losses of stocks and life history variants. Our data provided no direct evidence that large-scale genetic changes are tied to any recent historical events, although empirical sampling of genetic data near these events was not possible. Further, haplotype distributions did not indicate evidence for climate-mediated migration over large timescales. To the best of our knowledge, this study provides the first empirical test of the long-standing hypothesis that Chinook salmon in the Columbia River basin have experienced losses in genetic diversity from pre-contact period.

## Supporting information

S1 Fig Photographic example of salmon vertebrae. Example of salmon vertebrae analyzed in this study. Image on left shows fully intact vertebrae, middle and right images are examples of fragmented, partial vertebrae. Samples shown here are from the Spokane River group.
(TIF)

S2 Fig Rarefaction curves. Rarefaction curves for ancient and contemporary samples from the Columbia, Spokane and Snake River sample groups. Spokane samples are compared to Columbia subgroups as a proxy for single stock comparisons. Columbia and Spokane River groups indicate sampling was likely maximized and distinct differences in the expected number of haplotypes for ancient and contemporary samples, whereas the Snake River group indicates similarity in expected number of haplotypes for ancient and contemporary samples.
(TIF)

S1 Table Contemporary samples. Location, run timing, and collection year(s) for contemporary Chinook Salmon in the study. The Columbia River group is organized by tributary and the Snake River group by genetic stock identification reporting group. (*Data from Martin et al. [42]).
(PDF)

S2 Table Ancient sample summary. Sample ages, extraction data, species identification, and control region haplotype results for all ancient samples described in the study. PCR method indicates the method that which generated amplifiable DNA.
(PDF)

S3 Table Exact test p-values. Raw p-values for exact tests of population differentiation. Priest Rapids Hatchery is not included in the "Contemporary" group for the Columbia River as it is not a Grand Coulee Fish Maintenance (GCFMP) redirection sub-population.
(PDF)

S4 Table Primer data. Sequence, position, and annealing temperature for primer sets used to determine haplotype. Sequences are listed 5′—3′ with nucleotide positions relative to NCBI reference sequence NC_298.
(PDF)

S1 Text Samples. Detailed descriptions of sample context.
(DOCX)

S2 Text Methods. Detailed descriptions of DNA extraction and amplification from ancient and contemporary samples.
(DOCX)
