# Supplementary figures and images for: Increased mitochondrial DNA diversity in ancient Columbia River basin Chinook salmon Oncorhynchus tshawytscha
Source: PLoS One. 2018 Jan 10;13(1):e0190059. doi: 10.1371/journal.pone.0190059 (PMC5761847; doi:10.1371/journal.pone.0190059)

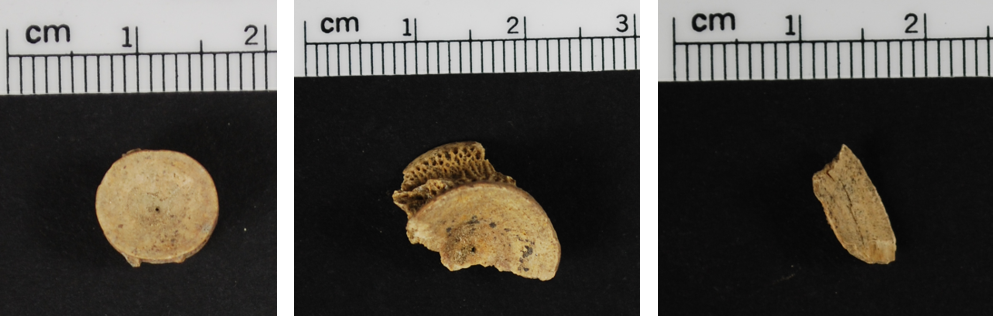

Supplement: S1 Fig — Example of salmon vertebrae analyzed in this study. Image on left shows fully intact vertebrae, middle and right images are examples of fragmented, partial vertebrae. Samples shown here are from the Spokane River group. (TIF) [file pone.0190059.s001.tif]

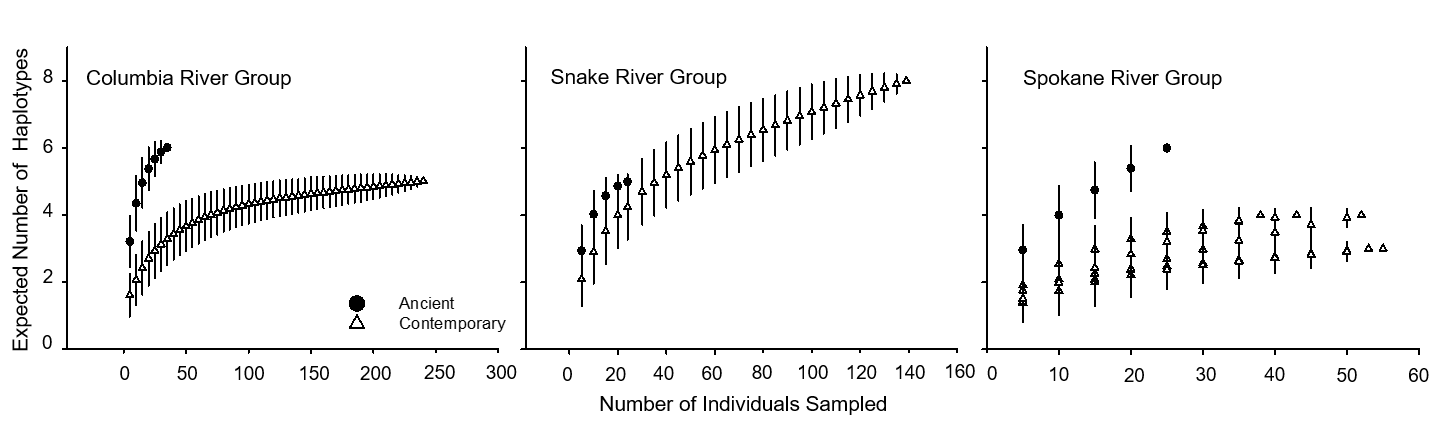

Supplement: S2 Fig — Rarefaction curves for ancient and contemporary samples from the Columbia, Spokane and Snake River sample groups. Spokane samples are compared to Columbia subgroups as a proxy for single stock comparisons. Columbia and Spokane River groups indicate sampling was likely maximized and distinct differences in the expected number of haplotypes for ancient and contemporary samples, whereas the Snake River group indicates similarity in expected number of haplotypes for ancient and contemporary samples. (TIF) [file pone.0190059.s002.tif]
