# Supplementary material for: Increased mitochondrial DNA diversity in ancient Columbia River basin Chinook salmon Oncorhynchus tshawytscha
Source: PLoS One. 2018 Jan 10;13(1):e0190059. doi: 10.1371/journal.pone.0190059 (PMC5761847; doi:10.1371/journal.pone.0190059)
Supplement: S1 Table — Location, run timing, and collection year(s) for contemporary Chinook Salmon in the study. The Columbia River group is organized by tributary and the Snake River group by genetic stock identification reporting group. (*Data from Martin et al. [42]). (PDF) [file pone.0190059.s003.pdf]

S1 Table. Contemporary samples. Location, run timing, and collection year(s) for contemporary Chinook Salmon in the study. The Columbia River group is organized by tributary and the Snake River group by genetic stock identification reporting group. (*Data from Martin et al. [42])

			Haplotype: TSA___									
Location	Run timing	Collection year(s)	1A	1B	4A	10	12	17	18	19	22	25
Columbia River Group												
Carson and Leavenworth Hatchery Complex												
Carson Fish Hatchery (1)		1995	--	--	--	--	--	13	--	--	--	--
Entiat National Fish Hatchery (2,3)	Spring	2002 - 2005	--	--	--	3	--	13	--	--	--	--
Leavenworth National Fish Hatchery (2)	Spring	2008	--	--	--	--	--	4	--	--	--	--
Winthrop National Fish Hatchery (2)	Spring	2002 - 2010	--	1	--	1	--	20	--	--	--	--
Entiat												
Rotary Screw Trap (2)	Spring	2005	--	--	--	2	--	1	--	--	--	--
Spawning Ground Carcass Recovery (2)	Spring & Summer	2003 - 2005	--	--	--	2	--	13	--	--	--	--
Lower River Rotary Screw Trap (2)	Spring	2009	--	1	--	4	--	11	--	--	--	--
Entiat River natural (3)	Spring	2011	--	--	--	2	--	8	--	--	--	--
Entiat River smolt (1)	Spring	2002	--	--	--	--	--	9	--	--	--	--
Methow												
Spawning grounds (3)	Spring	<2011	--	--	--	--	--	21	--	--	3	--
Twisp weir, spawning grounds (3)	Spring	<2011	--	--	--	3	--	13	--	--	2	--
Wenatchee												
White River (2)	Spring	<2011	--	--	--	1	--	15	--	--	--	--
Dryden trap (2)	Summer	<2011	--	--	--	--	1	10	--	--	--	--
Chiwawa (1)	Spring	2006	--	1	--	--	--	9	--	--	--	--
Nason Creek (1)		2009	--	1	--	--	--	--	--	--	--	--
Icicle												
Icicle Creek (2)	Spring	2001 - 2010	--	1	--	2	--	48	--	--	1	--
Snake River Group												
Chamberlain												
Chamberlain Creek (4)	Spring/Summer	2003- 2009	--	--	--	--	--	6	--	--	--	--
Chamberlain Creek, west fork (4)	Spring/Summer	2003, 2009	--	--	--	--	--	4	--	--	--	--
Hells Canyon												
Catherine Creek (4)	Spring/Summer	2008 - 2010	2	--	--	--	--	7	--	--	--	1
Upper Grande Ronde (4)	Spring/Summer	2003 - 2009	1	--	--	--	--	9	--	--	--	--
Imnaha River (4)	Spring/Summer	1998, 2008	1	1	--	2	--	5	--	--	--	--
Lemhi												
Hayden Creek (4)	Spring/Summer	2003, 2009	--	--	--	--	--	3	--	--	--	--
Lemhi River, L3A Trap (4)	Spring/Summer	2009, 2010	1	--	--	--	--	4	--	--	--	--
Middle Fork Salmon River												
Bear Valley Creek (4)	Spring/Summer	2006, 2009	--	--	--	--	--	2	--	--	--	--
Big Creek (4)	Spring/Summer	1999 - 2005	--	--	--	--	--	3	--	--	--	--
Camas Creek (4)	Spring/Summer	2003- 2009	--	--	--	--	--	3	--	--	--	--
Cape Horn Creek (4)	Spring/Summer	2005 - 2009	1	--	--	--	--	2	--	--	--	--
Elk Creek (4)	Spring/Summer	2003 - 2006	--	--	--	--	--	3	--	--	--	--
Marsh Creek (4)	Spring/Summer	1989	--	--	--	--	--	1	--	--	--	--
Sulphur Creek (4)	Spring/Summer	2003, 2007	--	--	--	--	--	2	--	--	--	--
South Fork Salmon River												
Lake Creek (4)	Spring/Summer	2003 - 2010	1	--	--	--	--	6	--	--	--	--
Secesh River (4)	Spring/Summer	1989 - 2009	--	--	--	--	--	5	--	--	--	--
Upper Salmon												
East Fork Salmon River (4)	Spring/Summer	2004 - 2008	--	--	--	--	--	3	--	--	--	--
Pahsimeroi River (4)	Spring/Summer	2002 - 2012	--	--	--	--	--	5	--	--	--	--
Sawtooth weir, upper Salmon (4)	Spring/Summer	2003 - 2011	--	--	--	--	--	3	--	--	--	--
Upper Lemhi River (4)	Spring/Summer	2000 - 2010	--	--	--	--	--	3	--	--	--	--
Valley Creek (4)	Spring/Summer	1989 - 2003	--	--	--	--	--	2	--	--	--	1

Location	Run timing	Collection year(s)	Haplotype: TSA___									
			1A	1B	4A	10	12	17	18	19	22	25
Yankee Fork, West fork (4)	Spring/Summer	2000 - 2007	1	--	--	--	--	2	--	--	--	--
Tucannon												
Tucannon River (3)*	Spring/Summer	<2010	--	--	--	--	--	21	--	--	--	--
Other												
Lyons Ferry Hatchery (3)*	Fall	<2010	3	11	1	4	--	1	1	1	--	--

Source: (1) National Oceanic and Atmospheric Administration, (2) U.S. Fish and Wildlife Service, (3) Washington Department of Fish and Wildlife, (4) Idaho Department of Fish and Game
