# Supplementary material for: Increased mitochondrial DNA diversity in ancient Columbia River basin Chinook salmon Oncorhynchus tshawytscha
Source: PLoS One. 2018 Jan 10;13(1):e0190059. doi: 10.1371/journal.pone.0190059 (PMC5761847; doi:10.1371/journal.pone.0190059)
Supplement: S2 Table — Sample ages, extraction data, species identification, and control region haplotype results for all ancient samples described in the study. PCR method indicates the method that which generated amplifiable DNA. (PDF) [file pone.0190059.s004.pdf]

S2 Table. Ancient sample summary. Sample ages, extraction data, species identification, and DLoop haplotype results for all ancient samples described in the study. PCR method indicates the method that generated amplifiable DNA.

			DNA extraction			PCR and sequencing results		
Group: Site	Sample ID	Age (YBP)	#	Weight (mg) and method		PCR method	12S species (12S haplotype, Chinook salmon only)	Control region haplotype
Snake R.: 45WT41	3070-B	2450 - 4950	1	73	silica	none		
Snake R.: 45WT41	3151-C1	6650 - 7950	1	24	silica	none		
Snake R.: 45WT41	3419-C1-2Q8	6650 - 7950	1	7	silica	none		
Snake R.: 45WT41	3782-C1-2H6	6650 - 7950	1	37	silica	none		
Snake R.: 45WT41	3785-C1-2O5	6650 - 7950	1	43	silica	none		
Snake R.: 45WT41	3789-C1-2Q2	250 - 9950	1	46	silica	none		
Snake R.: 45WT41	3789-C1-2Q2	250 - 9950	2	152	p:c	none		
Snake R.: 45WT41	3805-C1-2M3	4950 - 6950	1	53	p:c	none		
Snake R.: 45WT41	3805-C1-2M3	4950 - 6950	2	75	silica	none		
Snake R.: 45WT41	4177-3	250 - 9950	1	288	p:c	rescue	*Catostomus spp.*	
Snake R.: 45WT41	4177-3	250 - 9950	2	42	silica	rescue		
Snake R.: 45WT41	4177-4	250 - 9950	1	30	silica	rescue	*Catostomus spp.*	
Snake R.: 45WT41	4177-6	250 - 9950	1	32	silica	rescue	*Catostomus spp.*	
Snake R.: 45WT41	4177-6	250 - 9950	2	195	p:c	standard		
Snake R.: 45WT41	4548-B-C2	1450 - 2950	1	198	p:c	standard	Chinook (type 1)	TSA10
Snake R.: 45WT41	4582-C-O3	6650 - 7950	1	11	silica	none		
Snake R.: 45WT41	4585-C-W2	6650 - 7950	1	14	silica	none		
Snake R.: 45WT41	4619-B-C10	2450 - 4950	1	78	p:c	standard	Chinook (type 1)	TSA01a
Snake R.: 45WT41	6360-B	6650 - 7950	1	78	silica	none		
Snake R.: 45WT41	6793-C-W2	250 - 9950	1	unk	silica	none		
Snake R.: 45WT41	6844-C-P9	250 - 9950	1	63	p:c	standard	Chinook (type 1)	Incomplete
Snake R.: 45WT41	6844-C-P9	250 - 9950	2	24	silica	none		
Snake R.: 45WT41	6925-C-C2	250 - 9950	1	83	silica	none		
Snake R.: 45WT41	6933-B-C6-1	250 - 9950	1	125	p:c	none		
Snake R.: 45WT41	6933-B-C6-1	250 - 9950	2	11	silica	none		
Snake R.: 45WT41	6933-B-C6-2	250 - 9950	1	75	p:c	standard	*Catostomus spp.*	
Snake R.: 45WT41	6933-B-C6-2	250 - 9950	2	33	silica	none		
Snake R.: 45WT41	6933-B-C6-4	250 - 9950	1	18	silica	rescue	*Catostomus spp.*	
Snake R.: 45WT41	6933-B-C6-5	250 - 9950	1	87	p:c	standard	*Catostomus spp.*	
Snake R.: 45WT41	6940-B-C5	250 - 9950	1	87	p:c	none		
Snake R.: 45WT41	6940-B-C5	250 - 9950	2	24	silica	none		
Snake R.: 45WT41	7005-2K4	250 - 9950	1	8	silica	none		
Snake R.: 45WT41	577	1450	1	52	silica	none		
Snake R.: 45FR40	833	1450	1	56	silica	none		
Snake R.: 45FR40	1202-1	1450	1	11	silica	none		
Snake R.: 45FR40	1202-2	1450	1	62	p:c	standard	Chinook (type 1)	TSA17
Snake R.: 45FR40	1202-2	1450	2	172	p:c	standard		
Snake R.: 45FR40	1202-2	1450	3	11	silica	standard		
Snake R.: 45FR40	1202-3	1450	1	329	p:c	none		
Snake R.: 45FR40	1202-3	1450	2	46	p:c	none		

Group: Site	Sample ID	Age (YBP)	DNA extraction #	Weight (mg) and method		PCR and sequencing results PCR method	12S species (12S haplotype, Chinook salmon only)	Control region haplotype
Snake R.: 45FR40	1202-3	1450	3	37	silica	none		
Snake R.: 45FR40	1202-4	1450	1	19	silica	none		
Snake R.: 45FR40	1202-5	1450	1	7	silica	none		
Snake R.: 45FR40	1202-6	1450	1	32	silica	none		
Snake R.: 45FR40	1202-7	1450	1	37	silica	standard	Chinook (type 1)	TSA23
Snake R.: 45FR40	1202-7	1450	2	78	p:c	rescue		
Snake R.: 45FR40	1202-7	1450	3	309	p:c	none		
Snake R.: 45FR40	1202-8	1450	1	84	silica	standard	Chinook (type 1)	TSA23
Snake R.: 45FR40	1202-8	1450	2	280	p:c	none		
Snake R.: 45FR40	1202-8	1450	3	132	p:c	none		
Snake R.: 45FR40	1212-F1	1450	1	7	silica	none		
Snake R.: 45FR40	2227-1559	450 - 3950	1	74	silica	none		
Snake R.: 45WT134	2273-1412	450 - 3950	1	63	silica	none		
Snake R.: 45WT134	2305-1578	450 - 3950	1	23	silica	none		
Snake R.: 45WT134	2354-1605	450 - 3950	1	50	silica	none		
Snake R.: 45WT134	2368-810	450 - 3950	1	54	silica	none		
Snake R.: 45WT134	2374-1430	450 - 3950	1	43	silica	none		
Snake R.: 45WT134	3399-781	450 - 3950	1	35	silica	none		
Snake R.: 45WT134	3401-350	450 - 3950	1	37	silica	none		
Snake R.: 45WT134	3402-422	450 - 3950	1	158	p:c	rescue	Chinook (type 1)	TSA26
Snake R.: 45WT134	3402-422	450 - 3950	2	41	silica	rescue		
Snake R.: 45WT134	3403-498	450 - 3950	1	15	silica	none		
Snake R.: 45WT134	3404-584	450 - 3950	1	280	p:c	standard	Chinook (type 1)	TSA17
Snake R.: 45WT134	3404-584	450 - 3950	2	41	silica	rescue		
Snake R.: 45WT134	3405-687	450 - 3950	1	242	p:c	standard	*Oncorhynchus kisutch*	
Snake R.: 45WT134	3405-687	450 - 3950	2	22	silica	rescue		
Snake R.: 45WT134	3406-919	450 - 3950	1	52	silica	none		
Snake R.: 45WT134	3408-1219	450 - 3950	1	45	silica	none		
Snake R.: 45WT134	3435-638	450 - 3950	1	80	silica	none		
Snake R.: 45FR50	3592-68.2	250 - 8950	1	11	silica	none		
Snake R.: 45FR50	5182-68.2	250 - 8950	1	33	silica	none		
Snake R.: 45FR50	5198-68.6	250 - 8950	1	73	silica	none		
Snake R.: 45FR50	5215-68.2	250 - 8950	1	21	silica	none		
Snake R.: 45FR50	6068-68.6	250 - 8950	1	58	silica	none		
Snake R.: 45FR50	6571-68.0	250 - 8950	1	87	silica	none		
Snake R.: 45FR50	6599-68.0	250 - 8950	1	20	silica	none		
Snake R.: 45FR50	9199-68.3-1	250 - 8950	1	24	silica	none		
Snake R.: 45FR50	9199-68.3-3	250 - 8950	1	46	p:c	none		
Snake R.: 45FR50	9199-68.3-3	250 - 8950	2	84	silica	none		
Snake R.: 45FR50	9394-68.1	250 - 8950	1	34	silica	none		
Snake R.: 45FR50	9395-68.4	250 - 8950	1	31	p:c	none		
Snake R.: 45FR50	9395-68.4	250 - 8950	2	22	silica	none		
Snake R.: 45FR50	9401-64.0	250 - 8950	1	143	silica	none		

			DNA extraction			PCR and sequencing results		
Group: Site	Sample ID	Age (YBP)	#	Weight (mg) and method		PCR method	12S species (12S haplotype, Chinook salmon only)	Control region haplotype
Snake R.: 45FR50	9423-63.0	250 - 8950	1	191	p:c	none		
Snake R.: 45FR50	9423-63.0	250 - 8950	2	54	silica	none		
Snake R.: 45FR50	9423-63.0	250 - 8950	3	144	p:c	none		
Snake R.: 45FR50	9423-63.0	250 - 8950	4	108	p:c	none		
Snake R.: 45FR50	9434-62.22	250 - 8950	1	21	silica	none		
Snake R.: 45FR50	9440-64.184-1	250 - 8950	1	23	silica	none		
Snake R.: 45FR50	9440-64.184-2	250 - 8950	1	29	silica	none		
Snake R.: 45FR50	9444-62.16	250 - 8950	1	52	silica	none		
Snake R.: 45FR50	9494-62.10	250 - 8950	1	31	silica	none		
Snake R.: 45FR50	9520-62.19-1	250 - 8950	1	37	silica	none		
Snake R.: 45FR50	9520-62.19-2	250 - 8950	1	54	silica	none		
Snake R.: 45FR39	53-535	250 - 2950	1	167	silica	none		
Snake R.: 45FR39	57-516	250 - 2950	1	96	p:c	standard	Chinook (type 4)	TSA23
Snake R.: 45FR39	57-516	250 - 2950	2	89	p:c	none		
Snake R.: 45FR39	57-516	250 - 2950	3	52	silica	none		
Snake R.: 45FR39	334-515	250 - 2950	1	139	p:c	standard	*Ptychocheilus spp.*	
Snake R.: 45FR39	334-515	250 - 2950	2	66	silica	none		
Snake R.: 45FR39	334-515	250 - 2950	3	79	p:c	none		
Snake R.: 45FR39	341-438	250 - 2950	1	85	p:c	standard	*Oncorhynchus kisutch*	
Snake R.: 45FR39	341-438	250 - 2950	2	135	silica	none		
Snake R.: 45FR39	536-770-1	250 - 2950	1	42	silica	standard	Chinook (type 1)	TSA17
Snake R.: 45FR39	536-770-2	250 - 2950	1	48	silica	standard	Chinook (type 1)	TSA17
Snake R.: 45FR39	704-602	250 - 2950	1	32	silica	none		
Snake R.: 45FR39	813-742	250 - 2950	1	52	silica	none		
Snake R.: 45FR39	813-742	250 - 2950	2	62	p:c	none		
Snake R.: 45FR39	903-235	250 - 2950	1	31	silica	none		
Snake R.: 45FR39	1253-15	250 - 2950	1	87	silica	none		
Snake R.: 45FR39	1253-15	250 - 2950	2	280	p:c	none		
Snake R.: 45FR39	1344-449	250 - 2950	1	28	silica	none		
Snake R.: 45FR39	1387-273	250 - 2950	1	48	silica	none		
Snake R.: 45FR39	1513-658	250 - 2950	1	75	silica	none		
Snake R.: 45FR39	1513-658	250 - 2950	2	58	p:c	none		
Snake R.: 45FR39	1634-796	250 - 2950	1	46	p:c	none		
Snake R.: 45FR39	1634-796	250 - 2950	2	23	silica	none		
Snake R.: 45FR39	1634-796	250 - 2950	3	27	p:c	none		
Snake R.: 45FR39	1731-450-1	250 - 2950	1	57	silica	none		
Snake R.: 45FR39	2181-637	250 - 2950	1	27	silica	rescue	*Catostomus spp.*	
Snake R.: 45FR39	2379-368	250 - 2950	1	38	silica	rescue	*Ptychocheilus spp.*	
Snake R.: 45FR39	2602-679-1	250 - 2950	1	85	silica	none		
Snake R.: 45FR39	2602-679-2	250 - 2950	1	23	silica	none		
Snake R.: 45FR39	2730-316	250 - 2950	1	31	silica	none		
Snake R.: 45FR39	2764-466-2	250 - 2950	1	54	silica	none		
Snake R.: 45FR39	2834-540	250 - 2950	1	70	p:c	none		

			DNA extraction			PCR and sequencing results		
Group: Site	Sample ID	Age (YBP)	#	Weight (mg) and method		PCR method	12S species (12S haplotype, Chinook salmon only)	Control region haplotype
Snake R.: 45FR39	2834-540	250 - 2950	2	110	silica	none		
Snake R.: 45FR39	2834-540	250 - 2950	3	415	p:c	none		
Snake R.: 45FR39	3068-474-1	250 - 2950	1	30	silica	standard	*Ptychocheilus spp.*	
Snake R.: 45FR39	3327-350	250 - 2950	1	144	p:c	standard	Chinook (type 1)	TSA17
Snake R.: 45FR39	3327-350	250 - 2950	2	35	silica	standard		
Snake R.: 45FR39	3355-95	250 - 2950	1	140	p:c	none		
Snake R.: 45FR39	3355-95	250 - 2950	2	57	silica	none		
Snake R.: 45FR39	3362-93	250 - 2950	1	44	silica	none		
Snake R.: 45FR39	3364-134-1	250 - 2950	1	27	silica	none		
Snake R.: 45FR39	3371-37	250 - 2950	1	33	silica	standard	Chinook (type 1)	TSA10
Snake R.: 45FR39	3371-37	250 - 2950	2	168	p:c	standard		
Snake R.: 45FR39	3389-61	250 - 2950	1	26	silica	standard	Ptychocheilus spp.	
Snake R.: 45FR39	3408-80	250 - 2950	1	44	silica	none		
Snake R.: 45FR39	3417-66	250 - 2950	1	201	p:c	none		
Snake R.: 45FR39	3417-66	250 - 2950	2	46	silica	none		
Snake R.: 45FR39	3440-11	250 - 2950	1	85	p:c	none		
Snake R.: 45FR39	3440-11	250 - 2950	2	60	silica	none		
Snake R.: 45FR39	3444-153	250 - 2950	1	83	p:c	standard	*Ptychocheilus spp.*	
Snake R.: 45FR39	3444-153	250 - 2950	2	37	silica	none		
Snake R.: 45FR39	4554-246	250 - 2950	1	49	silica	none		
Snake R.: 45FR39	4556-485-1	250 - 2950	1	30	silica	standard	*Catostomus spp.*	
Snake R.: 45FR39	4556-485-2	250 - 2950	1	32	silica	standard	*Catostomus spp.*	
Snake R.: 45GA61	6071-0	650 - 950	1	73	p:c	standard	Chinook (type 1)	TSA01a
Snake R.: 45GA61	9015-0	650 - 950	1	233	p:c	standard	Chinook (type 1)	TSA17
Snake R.: 45GA61	9800-0	450 - 7950	1	265	p:c	standard	*Catostomus spp.*	
Snake R.: 45GA61	9800-0	450 - 7950	2	19	silica	none		
Snake R.: 45GA61	11134-54	650 - 950	1	241	p:c	standard	Chinook (type 1)	TSA10
Snake R.: 45GA61	11135-59	650 - 950	1	412	p:c	rescue	Chinook (type 1)	TSA17
Snake R.: 45GA61	11175-30	650 - 950	1	64	p:c	standard	Human (contamination)	
Snake R.: 45GA61	11175-30	650 - 950	2	34	silica	none		
Snake R.: 45GA61	11197-68	450 - 7950	1	23	silica	none		
Snake R.: 45GA61	11920-54	650 - 950	1	315	p:c	standard	Chinook (type 1)	TSA17
Snake R.: 45GA61	11920-54	650 - 950	2	25	silica	standard		
Snake R.: 45GA61	11924-51	450 - 7950	1	31	silica	standard	*Catostomus spp.*	
Snake R.: 45GA61	11957-123	450 - 7950	1	44	p:c	standard	*Catostomus spp.*	
Snake R.: 45GA61	11957-123	450 - 7950	2	32	silica	none		
Snake R.: 45GA61	11957-123	450 - 7950	3	85	p:c	none		
Snake R.: 45GA61	12206-68	650 - 950	1	84	p:c	standard	Chinook (type 1)	TSA17
Snake R.: 45GA61	12224-30	650 - 950	1	329	p:c	standard	Chinook (type 1)	TSA17
Snake R.: 45GA61	12227-0	450 - 7950	1	38	silica	none		
Snake R.: 45GA61	12248-30	650 - 950	1	37	p:c	standard	*Catostomus spp.*	
Snake R.: 45GA61	12248-30	650 - 950	2	37	silica	none		
Snake R.: 45GA61	12248-30	650 - 950	3	134	p:c	none		

			DNA extraction			PCR and sequencing results		
Group: Site	Sample ID	Age (YBP)	#	Weight (mg) and method		PCR method	12S species (12S haplotype, Chinook salmon only)	Control region haplotype
Snake R.: 45GA61	12250-68	650 - 950	1	11	silica	none		
Snake R.: 45GA61	12252-55	650 - 950	1	49	p:c	none		
Snake R.: 45GA61	12252-55	650 - 950	2	32	silica	none		
Snake R.: 45GA61	12257-30	650 - 950	1	21	silica	standard	Chinook (type 1)	TSA17
Snake R.: 45GA61	12270-59	650 - 950	1	437	p:c	standard	Chinook (type 1)	TSA01a
Snake R.: 45FR46	79-0	5450 - 7450	1	7	silica	none		
Snake R.: 45FR46	117-0-1	250 - 4450	1	14	silica	none		
Snake R.: 45FR46	117-0-2	250 - 4450	1	25.4	silica	standard	Chinook (type 4)	TSA01a
Snake R.: 45FR46	117-0-3	250 - 4450	1	34	silica	none		
Snake R.: 45FR46	220-0	250 - 8950	1	unk	silica	none		
Snake R.: 45FR46	220-0-2	250 - 8950	1	28	p:c	none		
Snake R.: 45FR46	220-0-2	250 - 8950	2	36	silica	none		
Snake R.: 45FR46	539-0	250 - 8950	1	60	p:c	none		
Snake R.: 45FR46	539-0	250 - 8950	2	15	silica	none		
Snake R.: 45FR46	818-0	250 - 4450	1	28	silica	none		
Snake R.: 45FR46	979-0-1	250 - 4450	1	20	silica	none		
Snake R.: 45FR46	979-0-2	250 - 4450	1	22	silica	none		
Snake R.: 45FR46	979-0-3	250 - 4450	1	25	silica	none		
Snake R.: 45FR46	979-0-4	250 - 4450	1	26	silica	none		
Snake R.: 45FR46	979-0-5	250 - 4450	1	35	silica	none		
Snake R.: 45FR46	979-0-6	250 - 4450	1	40	silica	none		
Snake R.: 45FR46	979-0-7	250 - 4450	1	253	p:c	none		
Snake R.: 45FR46	1088-0	250 - 4450	1	47	silica	none		
Snake R.: 45FR46	1120-0	250 - 8950	1	57	p:c	none		
Snake R.: 45FR46	1120-0	250 - 8950	2	49	silica	none		
Snake R.: 45FR46	1130-0-1	250 - 4450	1	201	p:c	standard	*Ptychocheilus spp.*	
Snake R.: 45FR46	1130-0-2	250 - 4450	1	15	silica	none		
Snake R.: 45FR46	1130-0-3	250 - 4450	1	18	silica	none		
Snake R.: 45FR46	1130-0-4	250 - 4450	1	24	silica	none		
Snake R.: 45FR46	1130-0-5	250 - 4450	1	26	silica	none		
Snake R.: 45FR46	1152-0-1	250 - 4450	1	46	p:c	none		
Snake R.: 45FR46	1152-0-1	250 - 4450	2	31	p:c	none		
Snake R.: 45FR46	1152-0-2	250 - 4450	1	52	p:c	standard	*Catostomus spp.*	
Snake R.: 45FR46	1152-0-2	250 - 4450	2	8	silica	none		
Snake R.: 45FR46	1152-0-2	250 - 4450	3	60	p:c	none		
Snake R.: 45FR46	1152-0-3	250 - 4450	1	22	silica	none		
Snake R.: 45FR46	1152-0-3	250 - 4450	2	57	p:c	none		
Snake R.: 45FR46	1185-0	250 - 4450	1	348	p:c	standard	Chinook (type 1)	TSA17
Snake R.: 45FR46	1185-0	250 - 4450	2	37	silica	standard		
Snake R.: 45FR46	2901-0	250 - 4450	1	37	silica	none		
Snake R.: 45FR46	2932-0	250 - 4450	1	125	p:c	none		
Snake R.: 45FR46	2932-0	250 - 4450	2	64	silica	none		
Snake R.: 45FR46	3095-01	250 - 4450	1	60	p:c	standard	*Ptychocheilus spp.*	

			DNA extraction			PCR and sequencing results		
Group: Site	Sample ID	Age (YBP)	#	Weight (mg) and method		PCR method	12S species (12S haplotype, Chinook salmon only)	Control region haplotype
Snake R.: 45FR46	3095-02	250 - 4450	1	20	silica	none		
Snake R.: 45FR46	3095-03	250 - 4450	1	31	silica	none		
Snake R.: 45FR46	3095-03	250 - 4450	2	89	p:c	none		
Snake R.: 45FR46	3095-0-1	250 - 4450	1	21	silica	none		
Snake R.: 45FR46	3095-0-1	250 - 4450	2	46	p:c	none		
Snake R.: 45FR46	3095-0-9	250 - 4450	1	18	silica	rescue	*Ptychocheilus spp.*	
Snake R.: 45FR46	3095-0-10	250 - 4450	1	19	silica	none		
Snake R.: 45FR46	3095-0-11	250 - 4450	1	20	silica	none		
Snake R.: 45FR46	3095-0-11	250 - 4450	3	10	silica	none		
Snake R.: 45FR46	3095-0-2	250 - 4450	1	27	silica	rescue	*Catostomus spp.*	
Snake R.: 45FR46	3095-0-2b	250 - 4450	1	12	silica	none		
Snake R.: 45FR46	3095-0-3	250 - 4450	1	12	silica	none		
Snake R.: 45FR46	3095-0-4	250 - 4450	1	13	silica	none		
Snake R.: 45FR46	3095-0-5	250 - 4450	1	15	silica	none		
Snake R.: 45FR46	3095-0-6	250 - 4450	1	15	silica	none		
Snake R.: 45FR46	3095-0-7	250 - 4450	1	16	silica	none		
Snake R.: 45FR46	3095-0-8	250 - 4450	1	17	silica	none		
Snake R.: 45FR46	3095-0-12	250 - 4450	1	142	p:c	standard	*Catostomus spp.*	
Snake R.: 45FR46	3207-0-1	250 - 4450	1	57	p:c	standard	Human (contamination)	
Snake R.: 45FR46	3207-0-1	250 - 4450	2	126	silica	none		
Snake R.: 45FR46	3207-0-2	250 - 4450	1	45	silica	none		
Snake R.: 45FR46	3207-0-3	250 - 4450	1	26	silica	none		
Snake R.: 45FR46	3207-0-4	250 - 4450	1	38	silica	none		
Spokane R.: 45SP266	328-32-1	3250	1	387	p:c	none		
Spokane R.: 45SP266	328-32-2	3250	1	383	p:c	rescue	Chinook (type 1)	TSA10
Spokane R.: 45SP266	406-40-1	7200	1	18	silica	none		
Spokane R.: 45SP266	406-40-2	7200	1	56	p:c	none		
Spokane R.: 45SP266	458-48	2500	1	21	silica	none		
Spokane R.: 45SP266	458-48	2500	2	267	p:c	none		
Spokane R.: 45SP266	670-69	3250	1	16	silica	none		
Spokane R.: 45SP266	711-74	3250	1	unk	silica	none		
Spokane R.: 45SP266	711-74	3250	2	250	p:c	none		
Spokane R.: 45SP266	722-76-1	7200	1	84.0	p:c	standard	Chinook (type 1)	TSA01a
Spokane R.: 45SP266	722-76-1	7200	2	255	p:c	rescue		
Spokane R.: 45SP266	722-76-2	7200	1	17	silica	none		
Spokane R.: 45SP266	722-76-3	7200	1	182	p:c	none		
Spokane R.: 45SP266	722-76-4	7200	1	134.0	p:c	rescue	Chinook (type 1)	Incomplete
Spokane R.: 45SP266	722-76-5	7200	1	347.0	p:c	rescue	Chinook (type 1)	TSA27
Spokane R.: 45SP266	766-80-1	7200	1	144	p:c	none		
Spokane R.: 45SP266	766-80-2	7200	1	241.0	p:c	rescue	Chinook (type 1)	Incomplete
Spokane R.: 45SP266	766-80-3	7200	1	18	p:c	none		
Spokane R.: 45SP266	766-80-4	7200	1	12	silica	none		
Spokane R.: 45SP266	766-80-5	7200	1	109	p:c	none		

Group: Site	Sample ID	Age (YBP)	DNA extraction #	Weight (mg) and method		PCR and sequencing results PCR method	12S species (12S haplotype, Chinook salmon only)	Control region haplotype
Spokane R.: 45SP266	766-80-6	7200	1	22	p:c	none		
Spokane R.: 45SP266	766-80-7	7200	1	72	p:c	none		
Spokane R.: 45SP266	766-80-8	7200	1	415	p:c	none		
Spokane R.: 45SP266	766-80-9	7200	1	405.0	p:c	rescue	Chinook (type 1)	Incomplete
Spokane R.: 45SP266	816-88	2500	1	47	p:c	rescue	Chinook (type 1)	Incomplete
Spokane R.: 45SP266	816-88	2500	2	29	p:c	none		
Spokane R.: 45SP266	816-88	2500	3	13	silica	none		
Spokane R.: 45SP266	900-96-1	undetermined	1	30	p:c	none		
Spokane R.: 45SP266	900-96-2	undetermined	1	24.0	silica	rescue	Chinook (type 1)	TSA10
Spokane R.: 45SP266	900-96-3	undetermined	1	233.0	p:c	rescue	Chinook (type 1)	Incomplete
Spokane R.: 45SP266	909-97-1	7200	1	37	p:c	standard	Chinook (type 3)	Incomplete
Spokane R.: 45SP266	909-97-1	7200	2	32	p:c	none		
Spokane R.: 45SP266	909-97-2	7200	1	19.0	silica	rescue	Chinook (type 1)	Incomplete
Spokane R.: 45SP266	909-97-3	7200	1	29	p:c	none		
Spokane R.: 45SP266	1362-148	3250	1	19	silica	none		
Spokane R.: 45SP266	1362-148	3250	2	30	p:c	none		
Spokane R.: 45SP266	1371-587-1	3250 or 7200	1	19	p:c	none		
Spokane R.: 45SP266	1371-587-2	3250 or 7200	1	132.0	p:c	rescue	Chinook (type 1)	Incomplete
Spokane R.: 45SP266	1371-587-3	3250 or 7200	1	58.0	p:c	rescue	Chinook (type 1)	TSA17
Spokane R.: 45SP266	1371-587-4	3250 or 7200	1	30	p:c	none		
Spokane R.: 45SP266	1371-587-5	3250 or 7200	1	22	silica	none		
Spokane R.: 45SP266	1371-587-6	3250 or 7200	1	23	p:c	none		
Spokane R.: 45SP266	1371-587-7	3250 or 7200	1	63.0	p:c	rescue	Chinook (type 1)	TSA25
Spokane R.: 45SP266	1371-587-7	3250 or 7200	2	71.0	p:c	rescue		
Spokane R.: 45SP266	1371-587-8	3250 or 7200	1	170.0	p:c	rescue	Chinook (type 1)	TSA28
Spokane R.: 45SP266	1371-587-9	3250 or 7200	1	23	p:c	none		
Spokane R.: 45SP266	1375-149-2	7200	1	18	silica	none		
Spokane R.: 45SP266	1375-149-3	7200	1	26	p:c	none		
Spokane R.: 45SP266	1405-153-1	7200	1	38	p:c	none		
Spokane R.: 45SP266	1405-153-2	7200	1	20	silica	none		
Spokane R.: 45SP266	1405-153-3	7200	1	26	p:c	none		
Spokane R.: 45SP266	1405-153-4	7200	1	26	p:c	none		
Spokane R.: 45SP266	1405-153-5	7200	1	36	p:c	none		
Spokane R.: 45SP266	1405-153-6	7200	1	41	p:c	none		
Spokane R.: 45SP266	1405-153-7	7200	1	26	p:c	none		
Spokane R.: 45SP266	1405-153-8	7200	1	30	p:c	none		
Spokane R.: 45SP266	1421-154-1	7200	1	32	silica	none		
Spokane R.: 45SP266	1421-154-2	7200	1	33	p:c	none		
Spokane R.: 45SP266	1531-165	3250	1	167.0	p:c	rescue	Chinook (type 1)	TSA10
Spokane R.: 45SP266	1544-167	3250	1	34	silica	none		
Spokane R.: 45SP266	1544-167	3250	2	41	p:c	none		
Spokane R.: 45SP266	1548-168	3250	1	26.0	silica	rescue	Chinook (type 1)	TSA10
Spokane R.: 45SP266	1548-168	3250	2	63.0	p:c	rescue		

			DNA extraction			PCR and sequencing results		
Group: Site	Sample ID	Age (YBP)	#	Weight (mg) and method		PCR method	12S species (12S haplotype, Chinook salmon only)	Control region haplotype
Spokane R.: 45SP266	1562-170-1	3250 or 7200	1	14	silica	rescue	Chinook (type 3)	TSA17
Spokane R.: 45SP266	1562-170-1	3250 or 7200	2	26	silica	none		
Spokane R.: 45SP266	1562-170-1	3250 or 7200	3	42	p:c	none		
Spokane R.: 45SP266	1562-170-1	3250 or 7200	4	143.0	p:c	standard		
Spokane R.: 45SP266	1562-170-2	3250 or 7200	1	18.0	silica	rescue	Chinook (type 1)	TSA17
Spokane R.: 45SP266	1562-170-2	3250 or 7200	2	unk	p:c	rescue		
Spokane R.: 45SP266	1562-170-2	3250 or 7200	3	28	p:c	none		
Spokane R.: 45SP266	1566-171-1	3250 or 7200	1	66.0	p:c	rescue	Chinook (type 1)	TSA17
Spokane R.: 45SP266	1566-171-1	3250 or 7200	2	17	silica	none		
Spokane R.: 45SP266	1566-171-2	3250 or 7200	1	21	silica	standard	Chinook (type 1)	TSA17
Spokane R.: 45SP266	1566-171-2	3250 or 7200	2	123.9	p:c	rescue		
Spokane R.: 45SP266	1572-173	7200	1	16.7	silica	rescue	Chinook (type 1)	TSA17
Spokane R.: 45SP266	1572-173	7200	2	60.0	p:c	standard		
Spokane R.: 45SP266	1757-189	3250	1	39	p:c	none		
Spokane R.: 45SP266	1781-191-1	7200	1	74.0	p:c	standard	Chinook (type 1)	TSA17
Spokane R.: 45SP266	1781-191-2	7200	1	50	p:c	rescue	Chinook (type 1)	Incomplete
Spokane R.: 45SP266	1781-191-2	7200	2	19	silica	none		
Spokane R.: 45SP266	1957-209	3250	1	27	p:c	none		
Spokane R.: 45SP266	1964-210	undetermined	1	21	silica	none		
Spokane R.: 45SP266	1964-210	undetermined	2	49	p:c	none		
Spokane R.: 45SP266	2014-218	undetermined	1	75.0	p:c	rescue	Chinook (type 1)	Incomplete
Spokane R.: 45SP266	2014-218	undetermined	2	24	silica	none		
Spokane R.: 45SP266	2082-588-1	3250	1	25	silica	none		
Spokane R.: 45SP266	2082-588-2	3250	1	27	p:c	none		
Spokane R.: 45SP266	2082-588-3	3250	1	29	p:c	none		
Spokane R.: 45SP266	2138-231-1	7200	1	44	p:c	none		
Spokane R.: 45SP266	2138-231-2	7200	1	26	p:c	none		
Spokane R.: 45SP266	2179-236	2500	1	30	silica	none		
Spokane R.: 45SP266	2179-236	2500	2	28	p:c	none		
Spokane R.: 45SP266	2211-239	2500	1	140.0	p:c	rescue	Chinook (type 1)	TSA10
Spokane R.: 45SP266	2249-241	3250	1	46	silica	none		
Spokane R.: 45SP266	2249-241	3250	2	34	p:c	none		
Spokane R.: 45SP266	2260-242	3250	1	29	p:c	rescue	Chinook (type 1)	TSA10
Spokane R.: 45SP266	2260-242	3250	2	46.0	silica	none		
Spokane R.: 45SP266	2296-248-1	undetermined	1	38	p:c	rescue	Chinook (type 1)	Incomplete
Spokane R.: 45SP266	2296-248-1	undetermined	2	40.0	p:c	none		
Spokane R.: 45SP266	2296-248-2	undetermined	1	24	p:c	none		
Spokane R.: 45SP266	2296-248-2	undetermined	2	49	p:c	none		
Spokane R.: 45SP266	2296-248-3	undetermined	1	32	p:c	none		
Spokane R.: 45SP266	2296-248-3	undetermined	2	28	p:c	none		
Spokane R.: 45SP266	2296-248-5	undetermined	1	39	silica	none		
Spokane R.: 45SP266	2296-248-5	undetermined	2	34	p:c	none		
Spokane R.: 45SP266	2296-248-5	undetermined	3	40	p:c	none		

			DNA extraction			PCR and sequencing results		
Group: Site	Sample ID	Age (YBP)	#	Weight (mg) and method		PCR method	12S species (12S haplotype, Chinook salmon only)	Control region haplotype
Spokane R.: 45SP266	2296-248-6	undetermined	1	37	silica	none		
Spokane R.: 45SP266	2296-248-6	undetermined	2	33	p:c	none		
Spokane R.: 45SP266	2491-264	2500	1	18	silica	none		
Spokane R.: 45SP266	2491-264	2500	2	32	p:c	none		
Spokane R.: 45SP266	2515-269	undetermined	1	22	silica	none		
Spokane R.: 45SP266	2515-269	undetermined	2	41	p:c	none		
Spokane R.: 45SP266	2523-270-1	7200	1	34.5	silica	rescue	Chinook (type 2)	TSA17
Spokane R.: 45SP266	2523-270-1	7200	2	87.0	p:c	standard		
Spokane R.: 45SP266	2523-270-2	7200	1	53.0	p:c	rescue	Chinook (type 1)	TSA10
Spokane R.: 45SP266	2523-270-3	7200	1	82.0	p:c	standard	Chinook (type 1)	Incomplete
Spokane R.: 45SP266	2582-276	2500	1	15	silica	none		
Spokane R.: 45SP266	2667-285	3250	1	126.0	p:c	rescue	Chinook (type 1)	Incomplete
Spokane R.: 45SP266	2667-285	3250	1	17	silica	none		
Spokane R.: 45SP266	2693-289	2500	1	144.0	p:c	rescue	Chinook (type 1)	TSA01a
Spokane R.: 45SP266	2742-295-1	3250	1	44	p:c	none		
Spokane R.: 45SP266	2742-295-2	3250	1	32	p:c	none		
Spokane R.: 45SP266	2742-295-3	3250	1	64.0	p:c	none		
Spokane R.: 45SP266	2742-295-4	3250	1	9	silica	none		
Spokane R.: 45SP266	2742-295-5	3250	1	41	p:c	none		
Spokane R.: 45SP266	2742-295-6	3250	1	39	p:c	rescue	Chinook (type 1)	Incomplete
Spokane R.: 45SP266	2748-296-1	3250 or 7200	1	40.0	p:c	rescue	Chinook (type 1)	TSA25
Spokane R.: 45SP266	2748-296-1	3250 or 7200	2	unk	silica	none		
Spokane R.: 45SP266	2748-296-1	3250 or 7200	3	24.0	p:c	none		
Spokane R.: 45SP266	2748-296-2	3250 or 7200	1	228	p:c	rescue	Chinook (type 1)	Incomplete
Spokane R.: 45SP266	2748-296-3	3250 or 7200	1	23.1	silica	standard	Chinook (type 2)	Incomplete
Spokane R.: 45SP266	2754-297-1	7200	1	28	p:c	none		
Spokane R.: 45SP266	2754-297-1	7200	2	26	p:c	none		
Spokane R.: 45SP266	2754-297-2	7200	1	17	silica	none		
Spokane R.: 45SP266	2765-298-1	7200	1	34	silica	none		
Spokane R.: 45SP266	2765-298-1	7200	2	27	p:c	none		
Spokane R.: 45SP266	2765-298-2	7200	1	41	p:c	none		
Spokane R.: 45SP266	2867-307	2500	1	42	p:c	none		
Spokane R.: 45SP266	2916-313-2	3250 or 7200	1	121.4	p:c	standard	Chinook (type 1)	TSA25
Spokane R.: 45SP266	2916-313-3	3250 or 7200	1	58.0	silica	standard	Chinook (type 1)	TSA10
Spokane R.: 45SP266	2916-313-4	3250 or 7200	1	31	p:c	rescue	Chinook (type 1)	Incomplete
Spokane R.: 45SP266	3007-324	2500	1	19	silica	none		
Spokane R.: 45SP266	3007-324	2500	2	43	p:c	none		
Spokane R.: 45SP266	3202-349	7200	1	42	p:c	rescue	Chinook (type 1)	TSA10
Spokane R.: 45SP266	3240-354	3250	1	35	silica	none		
Spokane R.: 45SP266	3240-354	3250	2	48	p:c	none		
Spokane R.: 45SP266	3240-354	3250	3	41	p:c	none		
Spokane R.: 45SP266	3272-359	7200	1	64.0	p:c	rescue	Chinook (type 1)	TSA17
Spokane R.: 45SP266	3272-359	7200	2	121.0	p:c	rescue		

			DNA extraction			PCR and sequencing results		
Group: Site	Sample ID	Age (YBP)	#	Weight (mg) and method		PCR method	12S species (12S haplotype, Chinook salmon only)	Control region haplotype
Spokane R.: 45SP266	4119-443-1	2500	1	17	silica	none		
Spokane R.: 45SP266	4119-443-1	2500	2	42	p:c	none		
Spokane R.: 45SP266	4119-443-2	2500	1	38	p:c	none		
Spokane R.: 45SP266	4119-443-2	2500	2	27	p:c	none		
Spokane R.: 45SP266	5615-598	2500	1	23	silica	none		
Spokane R.: 45SP266	5658-614-1	undetermined	1	unk	silica	none		
Spokane R.: 45SP266	5658-614-2	undetermined	1	31.4	silica	rescue	Chinook (type 1)	Incomplete
Spokane R.: 45SP266	5658-614-2	undetermined	2	31	p:c	standard		
Spokane R.: 45SP266	5658-614-2	undetermined	3	38.0	p:c	none		
Spokane R.: 45SP266	5658-614-3	undetermined	1	24	p:c	none		
Spokane R.: 45SP266	5658-614-4	undetermined	1	43	p:c	none		
Spokane R.: 45SP266	5662-616-1	3250 or 7200	1	42	p:c	rescue	Chinook (type 1)	TSA10
Spokane R.: 45SP266	5662-616-2	3250 or 7200	1	42.1	silica	rescue	Chinook (type 1)	Incomplete
Spokane R.: 45SP266	5662-616-2	3250 or 7200	2	107.0	p:c	standard		
Spokane R.: 45SP266	5662-616-2	3250 or 7200	3	117.0	p:c	standard		
Columbia R.: 45ST97	183273-18019	100	1	330	p:c	none		
Columbia R.: 45ST97	183367-18297	100	1	530	p:c	none		
Columbia R.: 45ST97	183587-15148	100	1	19	p:c	none		
Columbia R.: 45ST97	183591-15260	100	1	46	p:c	standard	Chinook (type 1)	TSA01a
Columbia R.: 45DO189	3808	3127.5 +/- 132.5	1	103	p:c	standard	Chinook (type 4)	TSA01a
Columbia R.: 45DO189	3841	3127.5 +/- 132.5	1	unk	p:c	none		
Columbia R.: 45DO189	3746-1	3127.5 +/- 132.5	1	131	p:c	standard	Chinook (type 1)	TSA10
Columbia R.: 45DO189	3746-2	3127.5 +/- 132.5	1	137	p:c	standard	Chinook (type 1)	TSA17
Columbia R.: 45DO189	3746-3	3127.5 +/- 132.5	1	138	p:c	standard	Chinook (type 1)	TSA10
Columbia R.: 45DO189	3746-4	3127.5 +/- 132.5	1	117	p:c	standard	Chinook (type 1)	TSA01a
Columbia R.: 45DO189	3746-5	3127.5 +/- 132.5	1	94	p:c	standard	Chinook (type 1)	TSA24
Columbia R.: 45DO189	3746-6	3127.5 +/- 132.5	1	105	p:c	none		
Columbia R.: 45DO189	3746-7	3127.5 +/- 132.5	1	unk	p:c	none		
Columbia R.: 45DO189	3746-8	3127.5 +/- 132.5	1	unk	p:c	none		
Columbia R.: 45DO189	3758-1	3127.5 +/- 132.5	1	92	p:c	standard	Chinook (type 1)	TSA10
Columbia R.: 45DO189	3758-2	3127.5 +/- 132.5	1	112	p:c	standard	Chinook (type 1)	TSA10
Columbia R.: 45DO189	3758-3	3127.5 +/- 132.5	1	unk	p:c	none		
Columbia R.: 45DO189	3770-1	3127.5 +/- 132.5	1	113	p:c	standard	Chinook (type 1)	TSA10
Columbia R.: 45DO189	3770-2	3127.5 +/- 132.5	1	141	p:c	standard	Chinook (type 1)	TSA17
Columbia R.: 45DO189	3770-3	3127.5 +/- 132.5	1	109	p:c	standard	Chinook (type 1)	TSA01b
Columbia R.: 45DO189	3770-4	3127.5 +/- 132.5	1	121	p:c	standard	Chinook (type 1)	TSA17
Columbia R.: 45DO189	3770-4	3127.5 +/- 132.5	2	101	p:c	standard		
Columbia R.: 45DO189	3770-5	3127.5 +/- 132.5	1	163	p:c	standard	Chinook (type 1)	TSA17
Columbia R.: 45DO189	3770-6	3127.5 +/- 132.5	1	144	p:c	standard	Chinook (type 1)	TSA17
Columbia R.: 45DO189	3770-7	3127.5 +/- 132.5	1	121	p:c	standard	Chinook (type 1)	TSA17
Columbia R.: 45DO189	3770-8	3127.5 +/- 132.5	1	159	p:c	standard	Chinook (type 1)	TSA01b
Columbia R.: 45DO189	3770-9	3127.5 +/- 132.5	1	119	p:c	standard	Chinook (type 1)	TSA17
Columbia R.: 45DO189	3770-10	3127.5 +/- 132.5	1	131	p:c	standard	Chinook (type 1)	TSA01b

			DNA extraction			PCR and sequencing results		
Group: Site	Sample ID	Age (YBP)	#	Weight (mg) and method		PCR method	12S species (12S haplotype, Chinook salmon only)	Control region haplotype
Columbia R.: 45DO189	3770-11	3127.5 +/- 132.5	1	101	p:c	standard	Chinook (type 1)	TSA17
Columbia R.: 45DO189	3770-12	3127.5 +/- 132.5	1	153	p:c	standard	Chinook (type 1)	TSA17
Columbia R.: 45DO189	3770-13	3127.5 +/- 132.5	1	152	p:c	none		
Columbia R.: 45DO189	3770-14	3127.5 +/- 132.5	1	141	p:c	standard	Chinook (type 1)	TSA23
Columbia R.: 45DO189	3770-15	3127.5 +/- 132.5	1	151	p:c	standard	Chinook (type 1)	TSA01b
Columbia R.: 45DO189	3770-16	3127.5 +/- 132.5	1	118	p:c	standard	Chinook (type 1)	TSA17
Columbia R.: 45DO189	3770-17	3127.5 +/- 132.5	1	106	p:c	standard	Chinook (type 1)	TSA17
Columbia R.: 45DO189	3770-18	3127.5 +/- 132.5	1	115	p:c	standard	Chinook (type 1)	TSA01b
Columbia R.: 45DO189	3770-19	3127.5 +/- 132.5	1	36	p:c	none		
Columbia R.: 45DO189	3770-20	3127.5 +/- 132.5	1	38	p:c	standard	Chinook (type 1)	TSA10
Columbia R.: 45DO189	3770-21	3127.5 +/- 132.5	1	43	p:c	standard	Chinook (type 1)	TSA17
Columbia R.: 45DO189	3770-22	3127.5 +/- 132.5	1	105	p:c	standard	Chinook (type 3)	TSA01b
Columbia R.: 45FE45	7220	1150 +/- 50	1	127	p:c	none		
Columbia R.: 45FE45	7221	1150 +/- 50	1	141	p:c	standard	Chinook (type 1)	TSA01b
Columbia R.: 45FE45	7221	1150 +/- 50	2	13	p:c	standard		
Columbia R.: 45FE45	7274	1150 +/- 50	1	132	p:c	none		
Columbia R.: 45FE45	7356	1150 +/- 50	1	125	p:c	standard	Chinook (type 1)	TSA01b
Columbia R.: 45FE45	7356	1150 +/- 50	2	106	p:c	standard		
Columbia R.: 45FE45	7357	1150 +/- 50	1	166	p:c	standard	Chinook (type 1)	TSA01b
Columbia R.: 45FE45	7357	1150 +/- 50	2	120	p:c	standard		
Columbia R.: 45FE45	7358	1150 +/- 50	1	101	p:c	standard	Chinook (type 1)	TSA23
Columbia R.: 45FE45	7359	1150 +/- 50	1	unk	p:c	none		
Columbia R.: 45FE45	8747	1150 +/- 50	1	131	p:c	none		
Columbia R.: 45FE44	1855	7627 +/- 100	1	140	p:c	standard	Chinook (type 1)	TSA17
Columbia R.: 45FE44	1922	7627 +/- 100	1	98	p:c	rescue	Chinook (type 1)	Incomplete
Columbia R.: 45FE44	2014	7627 +/- 100	1	unk	p:c	none		
Columbia R.: 45FE44	2016	7627 +/- 100	1	unk	p:c	none		
Columbia R.: 45FE44	2079	7627 +/- 100	1	135	p:c	none		
Columbia R.: 45FE44	2314	7627 +/- 100	1	90	p:c	none		
Columbia R.: 45FE44	2314	7627 +/- 100	2	85	p:c	none		
Columbia R.: 45FE44	2577	7627 +/- 100	1	107	p:c	none		
Columbia R.: 45FE44	2577	7627 +/- 100	2	92	p:c	none		
Columbia R.: 45FE44	2746	7627 +/- 100	1	unk	p:c	none		
Columbia R.: 45FE44	2750	7627 +/- 100	1	unk	p:c	none		
