# Supplementary material for: Increased mitochondrial DNA diversity in ancient Columbia River basin Chinook salmon Oncorhynchus tshawytscha
Source: PLoS One. 2018 Jan 10;13(1):e0190059. doi: 10.1371/journal.pone.0190059 (PMC5761847; doi:10.1371/journal.pone.0190059)
Supplement: S3 Table — Raw p-values for exact tests of population differentiation. Priest Rapids Hatchery is not included in the "Contemporary" group for the Columbia River as it is not a Grand Coulee Fish Maintenance (GCFMP) redirection sub-population. (PDF) [file pone.0190059.s005.pdf]

S3 Table. Exact test *p* -values. Raw *p* -values for exact tests of population differentiation. Priest Rapids Hatchery is not included in the "Contemporary" group for the Columbia River as it is not a Grand Coulee Fish Maintenance (GCFMP) redirection sub-population.

			Columbia									Snake										
		Ancient Spokane	Ancient	Contemporary	Contemporary & Priest Rapids H.*	Carson and Leavenworth H.	Entiat	Icicle	Methow	Wenatchee	Priest Rapids H.* (non-GCFMP)	Ancient	Contemporary	Chamberlain	Grande Ronde	Imnaha River	Lemhi	Lyons Ferry H.*	MF Salmon R.	SF Salmon R.	Tucannon	Upper Salmon
	Ancient Spokane		0.09	0.00	0.00	0.00	0.00	0.00	0.00	0.00	0.21	0.03	0.00	0.01	0.00	0.48	0.08	0.00	0.00	0.01	0.00	0.00
Columbia	Ancient	0.09		0.00	0.00	0.00	0.00	0.00	0.00	0.00	0.32	0.05	0.00	0.04	0.00	0.88	0.19	0.01	0.00	0.03	0.00	0.00
	Contemporary	0.00	0.00		0.52	0.92	0.16	0.83	0.09	0.16	0.00	0.00	0.00	1.00	0.00	0.01	0.10	0.00	0.15	0.15	0.71	0.03
	Contemp. & Priest Rapids H.*	0.00	0.00	0.52		0.90	0.56	0.75	0.14	0.29	0.00	0.00	0.00	0.81	0.01	0.08	0.28	0.00	0.45	0.38	0.67	0.11
	Carson and Leavenworth H.	0.00	0.00	0.92	0.90		0.14	0.77	0.03	0.40	0.00	0.00	0.08	1.00	0.01	0.01	0.30	0.00	0.28	0.34	0.69	0.13
	Entiat	0.00	0.00	0.16	0.56	0.14		0.02	0.01	0.02	0.00	0.00	0.00	0.32	0.00	0.07	0.16	0.00	0.06	0.11	0.06	0.01
	Icicle	0.00	0.00	0.83	0.75	0.77	0.02		0.15	0.76	0.00	0.00	0.08	1.00	0.03	0.01	0.35	0.00	0.60	0.48	1.00	0.36
	Methow	0.00	0.00	0.09	0.14	0.03	0.01	0.15		0.03	0.00	0.00	0.00	0.61	0.01	0.03	0.28	0.00	0.13	0.24	0.13	0.05
	Wenatchee	0.00	0.00	0.16	0.29	0.40	0.02	0.76	0.03		0.00	0.00	0.37	1.00	0.04	0.04	0.47	0.00	0.70	0.60	0.80	0.36
	Priest Rapids H.* (non-GCFMP)	0.21	0.32	0.00	0.00	0.00	0.00	0.00	0.00	0.00		0.19	0.00	0.03	0.01	1.00	0.26	0.00	0.00	0.04	0.00	0.00
Snake	Ancient	0.03	0.05	0.00	0.00	0.00	0.00	0.00	0.00	0.00	0.19		0.00	0.12	0.11	0.61	0.66	0.00	0.05	0.20	0.00	0.02
	Contemporary	0.00	0.00	0.00	0.00	0.08	0.00	0.08	0.00	0.37	0.00	0.00		0.79	0.50	0.32	0.90	0.00	0.83	0.93	0.56	0.61
	Chamberlain	0.01	0.04	1.00	0.81	1.00	0.32	1.00	0.61	1.00	0.03	0.12	0.79		0.68	0.04	0.44	0.00	1.00	1.00	0.00	1.00
	Grande Ronde	0.00	0.00	0.00	0.01	0.01	0.00	0.03	0.01	0.04	0.01	0.11	0.50	0.68		0.09	1.00	0.00	0.61	1.00	0.05	0.80
	Imnaha River	0.48	0.88	0.01	0.08	0.01	0.07	0.01	0.03	0.04	1.00	0.61	0.32	0.04	0.09		0.57	0.03	0.04	0.14	0.00	0.04
	Lemhi	0.08	0.19	0.10	0.28	0.30	0.16	0.35	0.28	0.47	0.26	0.66	0.90	0.44	1.00	0.57		0.00	1.00	1.00	0.28	0.65
	Lyons Ferry H.*	0.00	0.01	0.00	0.00	0.00	0.00	0.00	0.00	0.00	0.00	0.00	0.00	0.00	0.00	0.03	0.00		0.00	0.00	0.00	0.00
	MF Salmon R.	0.00	0.00	0.15	0.45	0.28	0.06	0.60	0.13	0.70	0.00	0.05	0.83	1.00	0.61	0.04	1.00	0.00		1.00	0.44	1.00
	SF Salmon R.	0.01	0.03	0.15	0.38	0.34	0.11	0.48	0.24	0.60	0.04	0.20	0.93	1.00	1.00	0.14	1.00	0.00	1.00		0.36	1.00
	Tucannon	0.00	0.00	0.71	0.67	0.69	0.06	1.00	0.13	0.80	0.00	0.00	0.56	0.00	0.05	0.00	0.28	0.00	0.44	0.36		0.23
	Upper Salmon	0.00	0.00	0.03	0.11	0.13	0.01	0.36	0.05	0.36	0.00	0.02	0.61	1.00	0.80	0.04	0.65	0.00	1.00	1.00	0.23	

*Data from Martin et al. [42]
