# Supplementary material for: Increased mitochondrial DNA diversity in ancient Columbia River basin Chinook salmon Oncorhynchus tshawytscha
Source: PLoS One. 2018 Jan 10;13(1):e0190059. doi: 10.1371/journal.pone.0190059 (PMC5761847; doi:10.1371/journal.pone.0190059)
Supplement: S4 Table — Sequence, position, and annealing temperature for primer sets used to determine haplotype. Sequences are listed 5′—3′ with nucleotide positions relative to NCBI reference sequence NC_298. (PDF) [file pone.0190059.s006.pdf]

S4 Table. Primer data. Sequence, position, and annealing temperature for primer sets used to determine haplotype. Sequences are listed 5' - 3' with positions relative to NCBI reference sequence NC_2980.

Target position	Forward	Reverse	Annealing temperature (°C)
499-683	TCTTATTGCCCGTTACCCCC	TGATTCTTTATAGAATATCA	50
524-716	CCGGGCGTTCTCTATATATGC	CATAGTTCCCTGGAATTCAA	54
524-726	CCGGGCGTTCTCTATATATGC	TAACGGACCTTATGCACTTG	56
630-793	TTGAATTCCAGGGAACTATG	AAGGATCTTTCAGCGTAGGG	54
630-761	TTGAATTCCAGGGAACTATG	TTACCGCGCAGAAGCCGGGG	54
732-891	TCTAAGATTTCCCCGGCTTC	TGCCAAACTGCTATAAAGTGC	58
732-845	TCTAAGATTTCCCCGGCTTC	AAAACATCATGCTGATTTGA	50
816-1008	GCTTTAGTTAAGCTACGCCAG	CCAGGAAGTTTCAAATCAGCA	58
879-1054	TAGCAGTTTGGCACCGACAG	TGCTCGTGGGACTTTCTAGG	60
929-1077	GTAAAGTCAGGACCAAGCCTTT	GTATACATTAATAAACTTTCCG	54
953-1141	CCCCTAGCAACACCATTTTC	GGGATTAAGGGCATCCTCAC	58
962-1141	ACACCATTTTCCCGCCTAAC	GGGATTAAGGGCATCCTCAC	58
