# Supplementary material for: Increased mitochondrial DNA diversity in ancient Columbia River basin Chinook salmon Oncorhynchus tshawytscha
Source: PLoS One. 2018 Jan 10;13(1):e0190059. doi: 10.1371/journal.pone.0190059 (PMC5761847; doi:10.1371/journal.pone.0190059)
Supplement: S1 Text — Detailed descriptions of sample context. (DOCX) [file pone.0190059.s007.docx]

**S1 Text. Samples.** Detailed descriptions of sample context.

Columbia River group: Ancient

The Columbia River group contains samples from the Grand Coulee Dam Project Area (45DO189) (location 1 depicted in Fig 1), ones excavated from a single housepit and two yard blocks. The site experienced multiple occupations over the past 7000 years with up to 28 people in residence at any given time [1]. Fort Colvile (45ST97) (location 2 depicted in Fig 1) was a trading post that operated from 1825 to 1871 under the Hudson’s Bay Company [2]. The one sample successfully sequenced from this site represents an intermediate time point: ~AD 1850 AD. This temporal period occurs post-European contact but prior to that considered ‘contemporary’. The sample is omitted from selected analyses and such instances are noted. Kettle Falls (location 3 depicted in Fig 1) was an important Native American fishing site with evidence of extended and diverse occupation over 9000 years [3]. Samples were obtained from two archeological sites at Kettle Falls: Ksunku (45FE45) and Shonitkwu (45FE44). Shonitkwu (45FE44) was excavated in 1971 and 1974, however a large portion of records from the 1971 excavations were lost in a fire [4] so the materials and associated data are from the 1974 excavation [5]. Both the Fort Colvile site and Kettle Falls were inundated by Lake Roosevelt, congruent with completion of Grand Coulee Dam in 1941. Samples were excavated during periods of reservoir drawdown in the 1970s. These samples were provided courtesy of the Bureau of Reclamation and the Colville Confederated Tribes Repository, History/Archaeology Program.

Columbia River group: Contemporary

These samples were provided by the National Oceanic and Atmospheric Administration (NOAA), U.S. Fish and Wildlife Service (USFWS), and Washington Department of Fish and Wildlife (WDFW).

Snake River group: Ancient

The Snake River group contains samples from Windust Caves (45FR46) at Ice Harbor Dam (location 6 depicted in Fig 1), a series of nine caves distributed along approximately 2000 feet of cliff face on the Snake River [6]. The caves were likely used for shelter, as well as for storage, with artifacts dating as early as ~9000 YBP. Radiocarbon dates were not generated for this site and the Mazama ash layer, a calibration point for Columbia Plateau sites, is not present [6]. Therefore, samples from this site were dated exclusively via comparison to cultural sequences. Time points indicated in the sequence were correlated with the geological stratigraphy, as well as with other Columbia Plateau sites [6]. Two sites, Granite Point (45WT41) and Wexpusnime (45GA61), are associated with Lower Granite Dam (location 8 depicted in Fig 1). Granite Point appears to have been occupied, with intermittent hiatus, as a camp site over the past 10,000 years [7]. Evidence of both camp and house village occupations are noted in the Wexpusnime collection [8]. Three ancient house pit villages are located near Lower Monumental Dam (location 7 depicted in Fig 1): (1) Harder (45FR40, excavated 1957), (2) Hatiuhpuh (45WT134, excavated 1987 and 1989), and (3) Three Springs Bar (45FR39, excavated in 1961) are located near Lower Monumental Dam [9-11]. A fourth site, Marmes Rockshelter (45FR50) is also associated with Lower Monumental Dam. This site likely had human occupation as early as 11,000 YBP which lasted over 8000 years [12, 13]. These samples were provided by the Department of Anthropology at Washington State University who holds these collections in trust for the Walla Walla District United States Army Corps of Engineers under the provisions of 36CFR79 (Title 36, Chapter I of the Code of Federal Regulations, part 79).

Snake River group: Contemporary

The Snake River samples from Lyons Ferry Hatchery and Tucannon samples represent data collected by Martin et al. [14] and were provided by the Washington Department of Fish and Wildlife (WDFW) for that study. All remaining contemporary Snake River samples were collected and provided by the Idaho Department of Fish and Game (IDFG).

Spokane River group: Ancient only

The Spokane River collection had three components, approximated at 2500, 3250, and 7200 YBP [3, 15]. A portion of samples in this collection were discriminated from the 2500 YBP component but could not be placed definitively into either the 3250 or 7200 YBP units due to rodent disturbance (S. Walker, Department Archaeological and Historical Services EWU; *personal communication*). The Spokane River samples were obtained courtesy of the Spokane Tribe of Indians, the Eastern Washington University Department of Archaeological and Historical Services, and the City of Spokane.

References

1. Galm JR, Lyman RL. Archaeological Investigations at River Mile 590: The Excavations at 45DO189. Eastern Washington University Archaeological and Historical Services, 1988.

2. Barman J, Watson BM. Fort Colvile's Fur Trade Families and the Dynamics of Race in the Pacific Northwest. The Pacific Northwest Quarterly. 1999;90(3):140-53.

3. Galm JR. A design for management of cultural resources in the Lake Roosevelt basin of northeastern Washington: Archaeological and Historical Services, Eastern Washington University; 1994.

4. Chance DH, Chance JV. Kettle Falls: 1971 and 1974 Salvage Archaeology in Lake Roosevelt. 1982.

5. Pouley JO. Analysis of the Kettle Falls culture chronology temporal gaps. Archeology in Washington. 2008;14:3-20.

6. Rice HS. The cultural sequence at Windust Caves: Washington State University; 1965.

7. Leonhardy FC. Artifact Assemblages and Archaeological Units at Granite Point Locality 1 (45WT41), Southeastern Washington: Washington State University; 1970.

8. Nakonechny LD. Archaeological analysis of Area A, Wexpusnime site (45GA61)1998.

9. Brauner D. Archaeological data recovery at Hatiuhpuh, 45WT134, Whitman County, Washington: Department of Anthropology, Oregon State University; 1990.

10. Kenaston MR. The archaeology of the Harder Site, Franklin County, Washington. Washington State University Laboratory of Anthropology, 1966.

11. Daugherty RD, Purdy BA, Fryxell R. The descriptive archaeology and geochronology of the Three Springs Bar archaeological site, Washington. Washington State University Laboratory of Anthropology, 1967.

12. Hicks BA. Marmes Rockshelter: A final report on 11,000 years of cultural use: Washington State University; 2004.

13. Lyman RL. Human-behavioral and paleoecological implications of terminal Pleistocene fox remains at the Marmes Site (45FR50), eastern Washington state, USA. Quaternary Science Reviews. 2012;41:39-48.

14. Martin KE, Steele CA, Brunelli JP, Thorgaard GH. Mitochondrial variation and biogeographic history of Chinook salmon. Transactions of the American Fisheries Society. 2010;139(3):792-802. doi: 10.1577/t09-080.1. PubMed PMID: WOS:000277639200015.

15. Butler VL. Fish Remains from the Spokane Site (45SP266). Cheney, Washington: Eastern Washington University, 2006.
