# Supplementary material for: Increased mitochondrial DNA diversity in ancient Columbia River basin Chinook salmon Oncorhynchus tshawytscha
Source: PLoS One. 2018 Jan 10;13(1):e0190059. doi: 10.1371/journal.pone.0190059 (PMC5761847; doi:10.1371/journal.pone.0190059)
Supplement: S2 Text — Detailed descriptions of DNA extraction and amplification from ancient and contemporary samples. (DOCX) [file pone.0190059.s008.docx]

**S2 Text.** **Methods.** Detailed descriptions of DNA extraction and amplification from ancient and contemporary samples.

Ancient samples: DNA extractions

Samples were extracted in batches of seven with one accompanying extraction negative control. First, approximately 2 – 530 mg of bone was submerged in 6% (w/v) sodium hypochlorite (bleach) for 4 min [1] and the bleach decanted. The samples were then twice submerged in DNA-free water, with the water decanted following each submersion. Following these decontamination efforts, extractions utilized either a silica or phenol:chloroform (p:c) based procedure (S4 Table). Samples extracted via silica method were transferred to 1.5 mL tubes, to which aliquots of 500 μL of EDTA (ethylenediaminetetraacetic acid) were added, and gently rocked at room temperature for >48 hours. DNA was then extracted following the WSU method described by Cui et al. [2]. Samples extracted via p:c methods were transferred to 15 mL tubes, to which aliquots of 2mL of EDTA were added, and gently rocked at room temperature for >48 hours. DNA was extracted following a modified protocol of Kemp et al. [3] described in Moss et al. [4].

Ancient samples: Tests for amplification and inhibition

All extracts were initially tested for amplification and inhibition. Amplification was conducted with primers OST12S-F 5’-GCTTAAAACCCAAAGGACTTG-3’ and OST12S-R 5’- CTACACCTCGACCTGACGTT-3’ that target a 189 basepair (bp) portion of the 12S mitochondrial gene [5]. Note that originally [5] described the OST12S-R primer in the incorrect orientation. It has been corrected here. These primers have been demonstrated to be especially effective in amplifying salmonid mtDNA, however the sequences produced can be used to differentiate a variety of non-salmonid fish to the species level [5-8]. Specific PCR protocols are described below.

Inhibition tests followed Kemp et al. [8] (see schematic illustration in their Figure 1). In brief, PCRs were set-up with an aDNA control, one comprised of pooled DNA extracted from ~3500 year old northern fur seal (Callorhinus ursinus) remains [1, 9, 10]. This pool was created using individual DNA extracts previously verified to yield 181 base pair (bp) amplicons of northern fur seal mitochondrial cytochrome B gene using the following primers: CytB-F 5’-CCAACATTCGAAAAGTTCATCC-3’ and CytB-R 5’- GCTGTGGTGGTGTCTGAGGT-3’ (with an annealing temperature of 60°C) [11]. This control template DNA is then “spiked” with the DNA recovered from the salmonid vertebrae, that is, DNA to be tested for the presence of sufficient inhibition to prevent the northern fur seal mitochondrial DNA (mtDNA) from amplifying (and possibly that for fish mtDNA, which could explain false negatives). Note that the northern fur seal primers are incapable of amplifying salmonid mtDNA. One advantage of this approach to monitoring for the presence of PCR inhibitors is that the control is aDNA and exhibits characteristics common in ancient extracts (i.e., signatures of post-mortem chemical degradation, high levels of DNA fragmentation, and low concentrations) [1, 9, 10]. Another advantage, given that the degree of PCR inhibition is directly related to the size of DNA to be amplified [12], is that the northern fur seal mtDNA fragment size targeted by these reactions is similar to that targeted in salmonids (181 bp and 189 bp, respectively). Samples compromised by inhibition indicated were subjected to additional silica treatments as described in Kemp et al. [13] until either: (1) amplification was possible and inhibition was not indicated or (2) amplification was not possible but inhibition was not indicated. In the case of outcome one, amplifications were submitted for sequencing. In the case of outcome two, modified PCR methods were pursued (described below).

Ancient samples: PCR protocols

Polymerase selection was based on results from Monroe et al. [14] indicating Klentaq LA was the least susceptible of nine polymerase or polymerase blends to inhibition associated with DNA obtained from prehistoric salmonid vertebrae recovered from two archaeological sites in the Pacific Northwest (DgRv-003 and DgRv-006). Unmodified PCRs contained: 1X Omni Klentaq Reaction Buffer mix (containing a final concentration of MgCl_2_ at 3.5 mM), 0.32 mM dNTPs, 0.24 µM each of forward and reverse primer, 0.3 U of Omni Klentaq LA polymerase, and 1.5 µL of template DNA (for 15 µL PCR reaction volume). Reaction conditions consisted of an initial three-minute denaturation at 94°C, followed by sixty 15 s cycles of 94°C (denaturation), primer specific temperature (annealing), and 68°C (extension). This was followed with a final extension at 68°C for 3 minutes. Negative PCR controls and positive PCR controls (utilizing DNA extracted from contemporary Chinook salmon, added in the post-PCR lab prior to initiating PCR) accompanied all sets of standard PCRs and modified PCRs. Modified PCR protocols consisted of increasing DNA concentration 2X or 3X as well as the use of *rescue PCR* [15]. In brief, rescue PCR consists of increasing reagents in equal proportion while decreasing the volume of water to keep the reaction volume the same; rescue PCR treatments at +25% and +50% were utilized for the data presented here (S4 Table).

Ancient samples: Sequencing and species identification

Successful PCR amplification following all PCRs was confirmed via separation on a 4% agarose gel stained with ethidium bromide with approximate size determined against a 20 bp ladder (Bayou BioLabs). Amplicons were submitted for sequencing in both forward and reverse directions using the same primers utilized for amplification. Product clean-up and sequencing were performed by Molecular Cloning Laboratories (South San Francisco, CA). Sequences were aligned and analyzed using Sequencher v 4.8 (Gene Codes; Ann Arbor, MI). Sequences generated from the amplification test were compared to those provided by Jordan et al. [5] and to the NCBI nucleotide database using the Basic Local Alignment Search Tool (BLAST*)* to determine species.

Ancient samples: DLoop haplotype determination

Haplotypes were based on a 563 bp sequence of the mitochondrial genome, a region homologous to that previously sequenced from Chinook salmon throughout their range [16]. The region spans 414 bp of the 3’ portion of the control region (or DLoop), through the full 68 bp of the phenylalanine tRNA gene, and 81 bp of the 5’ portion of the 12S ribosomal RNA gene. Haplotype characterization was completed using overlapping targets of less-than 200 bp in length. Target position, primers, and annealing temperatures are given in S8 Table.

*Contemporary samples: DNA Extractions and DLoop haplotype determination*

Contemporary samples were rinsed in TE for 1 minute followed by brief submersion in DNA-free water. DNA digested via proteinase K was extracted following the p:c procedure of Sambrook et al. [17]. PCR protocol followed that described in Martin et al. [16] utilizing primers Forward: 5’-CCCGCCCCTGAAAGCCGAAT-3’, Reverse: 5’-CGTGCCCCCAGGTGCGTATG-3’ with an annealing temperature of 62°C. Amplification was confirmed via separation on a 1% agarose gel and approximate size was determined against a 1kb ladder (New England BioLabs). PCR was repeated one additional time for any failures. Successful amplifications were submitted for sequencing using a nested primer 5’-ATAACCGCGGTGGCTGGCAC-3’. Product clean-up and sequencing were performed by Molecular Cloning Laboratories (South San Francisco, CA). Sequences were aligned and analyzed using Sequencher v 4.8 (Gene Codes; Ann Arbor, MI).

References

1. Barta JL, Monroe C, Kemp BM. Further evaluation of the efficacy of contamination removal from bone surfaces. Forensic Science International. 2013;231(1):340-8.

2. Cui Y, Lindo J, Hughes CE, Johnson JW, Hernandez AG, Kemp BM, et al. Ancient DNA analysis of mid-holocene individuals from the Northwest Coast of North America reveals different evolutionary paths for mitogenomes. PLoS One. 2013;8(7):e66948.

3. Kemp BM, Malhi RS, McDonough J, Bolnick DA, Eshleman JA, Rickards O, et al. Genetic analysis of early Holocene skeletal remains from Alaska and its implications for the settlement of the Americas. American journal of physical anthropology. 2007;132(4):605-21.

4. Moss ML, Judd KG, Kemp BM. Can salmonids (Oncorhynchus spp.) be identified to species using vertebral morphometrics? A test using ancient DNA from Coffman Cove, Alaska. Journal of Archaeological Science. 2014;41:879-89.

5. Jordan LG, Steele CA, Thorgaard GH. Universal mtDNA primers for species identification of degraded bony fish samples. Molecular Ecology Resources. 2010;10(3):581-. doi: 10.1111/j.1755-0998.2010.02841.x. PubMed PMID: ISI:000276407300025.

6. Halffman CM, Potter BA, McKinney HJ, Finney BP, Rodrigues AT, Yang DY, et al. Early human use of anadromous salmon in North America at 11,500 y ago. Proceedings of the National Academy of Sciences. 2015;112(40).

7. Grier C, Flanigan K, Winters M, Jordan LG, Lukowski S, Kemp BM. Using Ancient DNA Identification and Osteometric Measures of Archaeological Pacific Salmon Vertebrae for Reconstructing Salmon Fisheries and Site Seasonality at Dionisio Point, British Columbia. Journal of Archaeological Science. 2013;40:544-55.

8. Kemp BM, Monroe C, Judd KG, Reams E, Grier C. Evaluation of methods that subdue the effects of polymerase chain reaction inhibitors in the study of ancient and degraded DNA. Journal of Archaeological Science. 2014;42:373-80.

9. Barta JL, Monroe C, Crockford SJ, Kemp BM. Mitochondrial DNA preservation across 3000 year old northern fur seal ribs is not related to bone density: Implications for forensic investigations. Forensic Science International 2014;239:11-8.

10. Winters M, Barta JL, Monroe C, Kemp BM. To clone or not to clone: method analysis for retrieving consensus sequences in ancient DNA samples. PLoS One. 2011;6(6):e21247.

11. Moss ML, Yang DY, Newsome SD, Speller CF, McKechnie I, McMillan AD, et al. Historical ecology and biogeography of North Pacific pinnipeds: Isotopes and ancient DNA from three archaeological assemblages. Journal of Island & Coastal Archaeology. 2006;1(2):165-90.

12. McCord B, Pionzio A, Thompson R. Analysis of the Effect of a Variety of PCR Inhibitors on the Amplification of DNA using RealTimePCR, MeltCurves and STR Analysis. U.S. Department of Justice, 2015.

13. Kemp BM, Monroe C, Smith DG. Repeat silica extraction: A simple technique for the removal of PCR inhibitors from DNA extracts. Journal of Archaeological Science. 2006;33(12):1680-9.

14. Monroe C, Grier C, Kemp BM. Evaluating the efficacy of various thermo-stable polymerases against co-extracted PCR inhibitors in ancient DNA samples. Forensic Science International. 2013;228(1):142-53.

15. Johnson BM, Kemp BM. Rescue PCR: Reagent-rich PCR improves amplification of degraded DNA extracts. Journal of Archaeological Science: Reports. *Accepted 2017*.

16. Martin KE, Steele CA, Brunelli JP, Thorgaard GH. Mitochondrial variation and biogeographic history of Chinook salmon. Transactions of the American Fisheries Society. 2010;139(3):792-802. doi: 10.1577/t09-080.1. PubMed PMID: WOS:000277639200015.

17. Sambrook J, Fritsch EF, Maniatis T. Molecular cloning: Cold spring harbor laboratory press New York; 1989.
